# Synthesis and Behavior of Hexamethylenetetramine-Based Ionic Liquids as an Active Ingredient in Latent Curing Formulations with Ethylene Glycol for DGEBA

**DOI:** 10.3390/molecules28020892

**Published:** 2023-01-16

**Authors:** Dawid Zielinski, Andrea Szpecht, Paulina Hinc, Marcin Smiglak

**Affiliations:** 1 Poznan Science and Technology Park, Adam Mickiewicz University Foundation, 61-612 Poznań, Poland; 2 Faculty of Chemistry, Adam Mickiewicz University in Poznań, 61-614 Poznań, Poland

**Keywords:** hexamethylenetetramine, ionic liquids, epoxy resin, ethylene glycol, curing agent, composites

## Abstract

The paper presents the preparation of new ionic liquids based on hexamethylenetetramine with bis(trifluoromethanesulfonyl)imide and dicyanamide anion, which were characterized in detail in terms of their purity (Ion Chromatography) and thermal properties (Differential Scanning Calorimetry), as well as stability. The obtained substances were used to develop curing systems with ethylene glycol, which were successfully tested for their application with bisphenol A diglycidyl ether molecule. In addition, the curing process and its relationship to the structure of the ionic liquid are characterized in detail. The research showed that hexamethylenetetramine-based new ionic liquids can be successfully designed using well-known and simple synthetic methods—the Delepine reaction. Moreover, attention was paid to their stability, related limitations, and the application of hexamethylenetetramine-based ionic liquids in epoxy-curing systems.

## 1. Introduction

It is commonly believed that ready-made composite materials are neutral to the environment and due to the lack of direct interactions at the molecular level, are not toxic [1]. This theorem is fulfilled for properly prepared composites, in which the network of connections between resins and the hardener are closed, well-known, predictable, and stable, and the resin itself does not contain solvents and other volatile organic compounds (VOCs) before the curing process [2].

The aim of this work was to provide new solution in the field of multifunctional initiating systems for the polymerization of epoxy resins. To be more precise, this solution is based directly on previously unknown ionic liquids, designed with well-known raw materials, reactions, and processes. The proposed cross-linking systems must be easy to prepare, stable and predictable in the context of their application and most importantly, well thought out. In addition, it is important to bring added value to the final products in the form of changing their mechanical properties and/or application potential through new functionalities. In the era of ecological awareness and social responsibility, an extremely important element of research is the use of substances for which the details of their environmental impact, toxicity and methods of obtainment are known. This is one of the ways to limit the emergence of new, unpredictable, and not fully tested substances in the industry, while still developing science and improving existing solutions, which this work was also intended to prove.

Ionic liquids (ILs) are chemical compounds with an ionic structure, consisting of a cation and an anion and having several specific properties. The cation of the ionic liquid is usually of organic origin and has a varied structure. On the other hand, anions of ionic liquids can be both organic and inorganic, and their nature significantly affects the properties of the entire compound. The most important feature of this group of compounds is undoubtedly an extremely wide range of applications, in which the following should be distinguished: (i) dissolution and processing of biomass [3,4,5], (ii) catalytic reactions [6,7,8], (iii) active ingredients in pharmaceutical preparations [9,10] or (iv) design of polymerizable monomers intended for modern material solutions [11,12,13]. The wide area of application of ionic liquids is due to their properties, among which the following should be mentioned: high thermal stability [14], high electrochemical window [15], low volatility [16] and flammability [17]. At the same time, it turns out that these compounds can successfully replace, among others, hardeners (as a latent curing agents) of epoxy resins [18,19,20,21] as well as a large group of property modifiers: (i) flame retardants [22,23,24], (ii) hydrophobizers [25,26] and (iii) compounds that increase the mechanical parameters of the composite [21,27,28,29,30].

However, despite so many literature reports and numerous confirming the unique applications of ionic liquids, there are still groups of these compounds that require further research. One of these understudied groups is the non-aromatic polycyclic amines with catalytic properties, which can potentially be used as structures of ionic liquid cations. The most important substances in this group include: (i) 1,4-diazabicyclo[2.2.2]octane (DABCO), (ii) 1,8-diazabicyclo[5.4.0]undec-7-ene (DBU), (iii) 1-azabicyclo[2.2.2]octane (ABCO), (iv) 1,5-diazabicyclo[4.3.0]non-5-ene (DBN) and hexamethylenetetramine (HMTA). The first two, DABCO and DBU, remain in the interest of many researchers, especially as new ionic liquids with catalytic properties [21]; DBN is not very popular due to its high similarity to DBU, while ABCO, because of its very high price, is practically not studied at all. In contrast, the simplest, best known and most easily available, as well as the cheapest polycyclic amine—HMTA, remains to this day the most unexplored source of ionic liquids, which may have a few unique properties, from catalytic, electrochemical and anti-corrosion to fuel applications. There are only a few reports of HMTA-based ionic liquids being obtained and used as components for the production of strong catalysts for obtaining hexahydroquinolines [31] and pyrido[2,3-d:5,6-d0]dipyrimidines [32]. In addition, it was confirmed that HMTA-ILs exhibit high ionic conductivity, which increases significantly with temperature [33]. A huge impact on the importance of HMTA in chemistry was the discovery of the Delepine reaction in 1895, which allows for a simple and easy synthesis of primary amines through the intermediate product, which is a quaternary ammonium derivative of HMTA. The key here is the fact that this intermediate product can be easily isolated and shows very high stability [34,35].

Another view is ethylene glycol (EG), which is a well-known, simple chemical compound that is constantly gaining new applications based on the excellent knowledge of the properties of this substance. Especially in combination with ILs there is an interesting set of reports about its applications as a microemulsifier [36,37], co-solvent [38,39,40] and a substance able to stabilize ionic liquids through a specific interaction [41].

All the literature reports cited above were an inspiration to conduct this research, the results of which will be presented in the following subsections.

## 2. Results

### 2.1. Synthesis of Ionic Liquids

**Figure 1:**
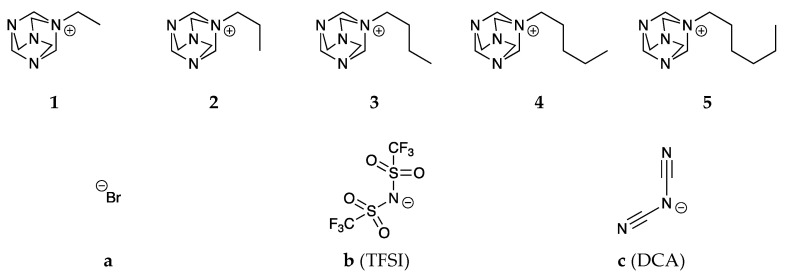
Chemical structures of the obtained compounds.

The research proposed a portfolio of new HMTA derivatives, differing in the length of the carbon chain attached to the nitrogen atom. Structures of all cations are shown in the Figure 1. As part of the research, alkyl chains with both an even and an odd number of carbon atoms were used to precisely determine the influence of the chain length on the properties of the obtained compounds. Moreover, two groups of anions were selected and combined with cation structures to obtain new ionic liquids. The bis(trifluoromethanesulfonyl)imide (TFSI) anion is highly hydrophobic [42], while the dicyanamide (DCA) anion is inherently more hydrophilic [43]. Such a correlation between anions allows for a thorough look at the nature of the compounds obtained and opens up more application possibilities and areas (mainly those indicated in the introduction) where new substances may prove value. To obtain the proposed substances, it was necessary to conduct quaternization reactions first, leading to the desired halogen derivatives, which were then subjected to metathesis reactions leading to the final products. Afterward, all obtained substances were subjected to a thorough analysis of physicochemical properties before they were subjected to application tests.

#### 2.1.1. Quaternization

The first step of the Delepine reaction is the attachment of an alkylated chain to the nitrogen atom, followed by a quaternary ammonium salt (QAS) of HMTA formation. The reaction proceeds at room temperature in chloroform, from which the product is easily separated by simple filtration. Thus, the process was highly efficient, and the products were recrystallized to obtain QASs of very high purity (Table 1). On the other hand, the obtained products do not show strong hygroscopicity in contrast to analogous derivatives for, e.g., DABCO [17]. Determination of the purity of the obtained substances was carried out using ion chromatography, considered to be the best method for determining the purity of ionic compounds [12,17,40,41,42]. In addition, the same technique was used to monitor the progress of the reaction. Through routine analysis of product increment and substrate depletion, the reaction time was precisely determined to enable high process yields to be obtained in the shortest possible time. As a result of the conducted experiments, five bromide derivatives were successfully obtained, reaching the efficiency exceeding 99% and the purity of the obtained substances at the average level of 99.2%. To fully identify the obtained compounds, they were subjected to NMR and mass spectrometry analysis. Another equally important value for ILs is their water content, especially for halides, which are a key step on the way for obtaining the desired further products. None of the salts obtained exceeded the level of 1% of water; moreover, compounds **1a**, **2a** and **5a**, did not exceed the level of 0.5% water. The lowest value was recorded for the derivative having a propyl chain (**2a**) in its structure.

#### 2.1.2. Metathesis Reaction

**Figure 2:**
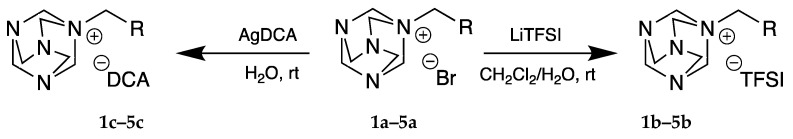
Scheme of metathesis reaction leading to HMTA derivatives with TFSI anion and DCA.

However, ten new TFSI and DCA ionic liquids were selected for research. The bromide salts obtained earlier were precursors on the way to their preparation and required further processing. For this purpose, two different pathways of the metathesis reaction were used between the bromide anion in the HMTA quaternary salt and (i) the dicyandiamide anion derived from the freshly prepared (according previously published work [21]) silver salt, or the (ii) bis(trifluoromethanesulfonyl)imide anion derived from lithium salt. Each pathway was analyzed at room temperature, however, using different process conditions, as shown in Figure 2, and synthetic techniques, as described in detail in the synthesis section. In the case of hydrophobic ionic liquids, the average yield of the metathesis reaction was 98.4%, while the purity of the obtained compounds ranged from 99.1 to 99.8%, with the lowest values for ionic liquids **4b** and **5b,** with the longest alkyl chains. Similar results were recorded for the synthesis of dicyandiamide derivatives, for which the average yield of the reaction was 94.5%, with the lowest values of 87.9% for compound **2c** and 93.9% for compound **4c**, for compounds with an odd number of carbon atoms in the alkyl chain. On the other hand, the purity of all dicyandiamide derivatives exceeded 97.5%, reaching the highest value of 98.1% for compounds **1c** and **3c** containing ethyl and butyl alkyl chains, respectively.

#### 2.1.3. Thermal Properties of the Compounds

Measurements of thermal properties of the obtained substances (thermograms from analyzes available in Supplementary Materials), show numerous differences between both individual groups of compounds (depending on the anion present in the IL structure) and depending on the length of the carbon chain, in particular the relationship between the even and odd number of carbon atoms in the alkyl chain (Table 2). Namely, for quaternary ammonium salts with a bromide anion, only an onset exothermic peak (T_decomp_) was observed in the thermogram, characteristic of the process of partial decomposition of the salt with the release of a large portion of energy. Differences in the decomposition temperature were observed, depending on whether an alkyl chain with an even or an odd number of carbon atoms was present in the structure. Compounds **2a** and **4a**, which contain a propyl and a pentyl chain structure, have onset peak decomposition temperatures of 146.5 °C and 143.6 °C, respectively. Between this group of compounds and analogs containing even carbon chains in the structure, an average temperature difference of 14 °C was observable. The highest decomposition temperatures, 168.3 °C and 155.4 °C, respectively, were recorded for compounds **1a** and **3a**, i.e., those with an ethyl or butyl chain. On the contrary, all ionic liquids obtained in the metathesis reaction, both DCA and TFSI, are characterized by lower crystallization/decomposition peak temperatures (T_decomp_) observed during the measurements. The lowest values were recorded for compounds **1b**, **4b**, **1c** and **3c** and amounted to the following: 119.3 °C, 121.6 °C, 120.3 °C and 101.6 °C, respectively. However, the highest values were for the derivatives **2b** (126.8 °C), **5b** (134.7 °C), **2c** (135.3 °C) and **5c** (126.8 °C). There was no clear relationship between the chain length and the parameters obtained. In addition, glass transition temperature (T_g_) data were collected for ILs with the TFSI anion, and melting point temperatures (T_m_) were collected for ILs with the DCA anion. For the glass transition temperature, there is a general shift towards lower temperatures with increasing chain length, resulting in the lowest temperature values for **4b** and **5b** being −29.1 °C and −19.9 °C, respectively. Similarly, the melting temperatures observed for the dicyanamides also show a shift towards higher temperatures with increasing chain length, except for compound **2c** (57.4 °C), which decreases relative to a compound **1c** (69.1 °C) with a shorter alkyl chain. All collected values are below 100 °C, reaching the highest value of 75.5 °C and 86.6 °C for **4c** and **5c** compounds, respectively.

### 2.2. Curing Process 

**Figure 3:**
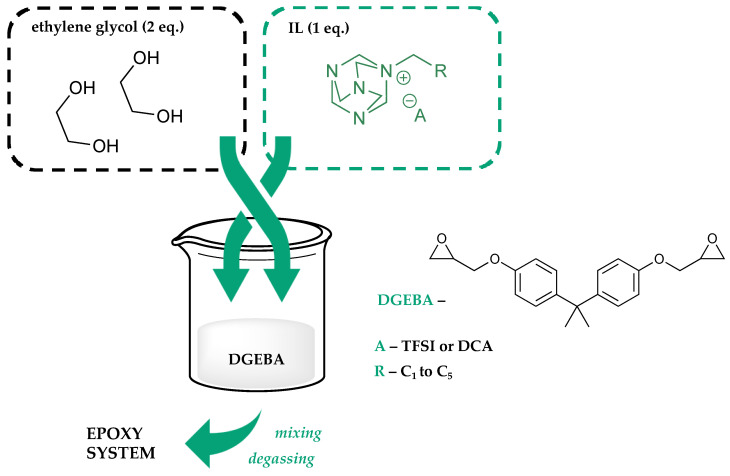
Scheme of the process of preparing resin systems with ionic liquids.

In the next stage of the research, a selected group of ten ILs was used to prepare ten curing mixtures intended for cross-linking the epoxy resin (Figure 3). The combinations were prepared by mixing the ionic liquid with ethylene glycol in a molar ratio of 1:2 in such a way as to ensure the highest possible curing activity in relation to the resin while maintaining high stability of the mixture itself. By experimenting with different ratios of IL to ethylene glycol, a system was selected in which there are two equivalents of EG for one equivalent of ionic liquid. Such a relationship between these substances resulted in the most stable mixtures, whose stability was validated by observing phase separation over a period of up to 60 days. Moreover, it was confirmed that ethylene glycol forms more stable systems with DCA-based ionic liquids, which successfully maintain their homogeneity beyond 60 days, while TFSI anion ionic liquids lose their homogeneity after about 2 weeks, requiring re-mixing. EG, due to its emulsifying properties, allowed us to prepare homogeneous curing systems with a high degree of compatibility with the resin, which in this case was the Bisphenol A diglycidyl ether (DGEBA, M = 340 g/mol) molecule. An important feature here is the fact that ethylene glycol creates much more stable emulsions in combination with more hydrophilic ionic liquids such as DCA ILs. It has been observed that TFSI causes emulsions to become heterogenous rather quickly. Specifically, to thoroughly study the curing process and compatibility, a system consisting of unmodified DGEBA molecules was used, thus eliminating the influence of possible modifiers and solvents that could be present in commercially available epoxy resins. Next, the prepared curing systems were mixed with a resin particle in a ratio that guaranteed full cross-linking (detailed data on sample preparation of resin systems are provided in the chapter Methods and in the reference [17]), degassed, and then cured in a vacuum oven with process temperature, developed based on data from simultaneously conducted tests using Differential Scanning Calorimetry (DSC). An important property of the curing mixtures is their initiating effect occurring only at elevated temperatures. At room temperature, they remain weakly active against the DGEBA molecule. In particular, this is a property observed with many ionic liquids used as curing initiators for epoxy resins and is fairly well explained [18,19,20,21].

#### 2.2.1. Thermal Analysis

Subsequently, ten curing systems were used to prepare mixtures with DGEBA, which were subjected to tests of curing process nature. For this purpose, Differential Scanning Calorimetry was again used (thermograms from analyzes available in Supplementary Materials), and several parameters of the process (Table 3) were determined, such as polymerization start temperature (T_onset_), temperature peak (T_max_) and polymerization end temperature (T_end_). At the same time, the amount of energy released (ΔH) during the process was monitored. In the case of curing systems based on ionic liquids with the TFSI anion, comparable results for individual parameters were obtained, which remained at a similar level of values, regardless of the type and length of the alkylated chain in the cation structure. The exception is the derivative with an ethyl chain (**1b**), for which a lower polymerization start temperature (109.4 °C) and a much higher process energy (84.4 J/g) were recorded. At the same time, for the polymerization initiated by these curing systems, the appearance of two independent areas of temperature peaks in the form of two thermal processes occurring in the sample was observed. The highest temperature values of the first thermal peak, respectively 154.4 °C and 149.0 °C, were obtained for systems containing compounds **3b** and **5b**, however, these differences are insignificant. Similarly, in the case of the second thermal peak, the highest values were recorded for systems containing compounds **2b** (224.4 °C) and **4b** (229.5 °C). Simultaneously, with the increase in the length of the alkyl chain in the cation structure, the amount of energy released during the second thermal process occurring in the sample decreases. On the other hand, polymerization initiated by systems based on dicyanamide ionic liquids proceeds in the form of a single thermal transformation process. In this case, the relationship between the polymerization starting temperature and the energy released during the process, dependent on an odd or even number of carbon atoms in the alkyl chain, is clearly visible. Thus, with an odd number of carbon atoms in the cation structure, relatively lower values of the process start temperature were observed, while the amount of released energy decreases with the increase in the length of the odd alkyl chain. In contrast, if there is an even number of carbon atoms in the alkyl chain of the ionic liquid, the energy of thermal transformation increases with its length, reaching a maximum of 151.5 J/g for the system containing compound **5c**, for which the lowest polymerization initiation temperature (101.3 °C) and polymerization peak temperature (151.8 °C) were also recorded. To summary, all characteristic temperature points of the cross-linking process are within the range of the decomposition temperature of ionic liquids.

#### 2.2.2. Hardness of the Cured Resin

Afterward, the prepared samples of the cured DGEBA were tested for the basic assessment of their hardness/brittleness depending on the curing system used. Such a quick experiment made it possible to determine the relationship between the structure of the IL and the behavior of the hardened material in laboratory conditions. Furthermore, the samples were evaluated 48 h after hardening and after 7 and 14 days to determine the effect of aging of the samples on the basic parameter, which was hardness. For comparison, analogous samples cured with two amines commonly used as initiators of epoxy resin polymerization, triethylenetetramine (TETA, RM1) and isophorone diamine (IPDA, RM2), whose hardening properties have already been thoroughly investigated [44], were made. The samples are tabulated and related to the results obtained for the new curing systems. With the passage of time, the changes in the hardness parameters of the samples did not change beyond 2D, therefore the Table 4 summarizes the collected data as average values for all measurements carried out for a given sample. The average value of the hardness of the samples in which systems with TFSI ILs were used was 79D, while systems with DCA IL was 85D and all these values are higher than the reference samples. The influence of the type of anion in the ionic liquid on the obtained hardness results is visible. DCA IL-based systems show a relatively higher hardness of the obtained materials than TFSI IL-based systems. However, in the case of these DCA systems, composed of ILs with an odd number of carbon atoms in the alkyl chain, they were characterized by significant brittleness, which made measurement impossible. Systems containing TFSI ILs have a smaller increase in hardness in relation to reference samples and at the same time, slightly higher values were observed for compounds **2b** (80D) and **4b** (81D), in which their structures contain chains with an odd number of carbon atoms.

## 3. Discussion and Conclusions

### 3.1. Synthesis and ILs Stability

Hexamethylenetetramine is an unusual amine, obtained by the reaction of formaldehyde and ammonia. It does not melt, only sublimates, and is very sensitive to any changes in pH. Improperly stored or contaminated, it slowly decomposes to formaldehyde and ammonia. The decomposition phenomenon is probably caused by the hygroscopicity of the amine itself and by the presence of water, initiating a slow hydrolysis process that leads to the formation of formaldehyde and ammonia [45]. On the other hand, under the influence of temperature or during combustion (with access to oxygen), the HMTA decomposition pathways change. To illustrate, thermal decomposition mainly leads to nitrogen oxides, carbon dioxide, carbon monoxide and formaldehyde, while the combustion process generates carbon dioxide, nitrogen, nitrogen oxides and trace amounts of ammonia. Based on studies of the kinetics of HMTA decay conducted to understand the behavior of this molecule in the human body, the following conclusions can be drawn: (i) HMTA solution in water has a pH of 9.5, (ii) pH values lower than 9.5 initiate the decomposition process, (iii) no release of formaldehyde is observed up to pH 7.4, (iv) at a pH around 2.0, formaldehyde is released in higher quantities [46]. We assume that during the thermal curing of the resin with HMTA-based ionic liquids, formaldehyde is not released due to the high process temperature, and carbon oxides are released in its place. Nevertheless, there is a risk that residual unreacted ionic liquid in the cured resin structure will be able to release formaldehyde during storage. This is not the subject of the presented research, but in the case of further experiments, it may be advisable to carry out tests in the direction of determination of volatile organic compounds (VOCs) from the cured material.

In this work, several new chemical compounds are presented. From our point of view, the key aspect to discuss here is undoubtedly the very high purity of the salts obtained and the high efficiency of the reactions. This shows how useful the first step of the Delepine reaction is for the synthesis of new potential ionic liquids. However, this reaction is limited to the amine substrate—HMTA. While the halide salts obtained directly in it show high stability, signs of lower stability appear in the case of metathesis reaction products. The spatial structure of the quaternary HMTA cation, especially as a product of only a single chain attachment, is likely to be energetically highly unstable. Moreover, the liquid form of the compound, which allows the migration of molecules and the presence of, for example, anions derived from strong acids, additionally affects the stability of the compounds obtained due to high impact on pH. As HMTA quaternary ammonium salts, the products are highly sensitive to any changes in pH that may occur through spontaneous partial decomposition of the cation, which will shift the ionic balance. In addition, the nature of ionic liquids favors the absorption of even small amounts of water, which is absorbed in the compounds and facilitates the hydrolysis of the compound. In the case of HMTA, this leads directly to the release of ammonia, the smell of which is observable in the case of TFSI and DCA ionic liquids over time of their storage at room temperature. Undoubtedly, this phenomenon is influenced primarily by the presence of water, but also by the fact that along with the breakdown of the cation structure, the concentration of anions increases, which results in a decrease in pH. In the case of the synthesis of quaternary ammonium salts based on HMTA, it is very important to monitor the purity of the obtained compounds, e.g., using ion chromatography. In this way, it is possible to quickly detect the potential presence of ammonia or other ionic structures that could be formed by the decomposition of the compound and, consequently, affect the possibility of using a given substance. On the one hand, the synthesis of this type of derivatives is exceptionally simple and gives a very wide field for the introduction of many alkylating agents from all groups of this type of compounds. However, one should be always carefully analyze the stability of the obtained substances and consider the possibility of changes in pH, which, by initiating the degradation process of the compound, may sometimes make it impossible to use them in specific application areas. In the case of application presented in this paper, the phenomenon of slow decay does not have a significant impact, and sometimes it is in favor of our solution.

### 3.2. Role of Ethylene Glycol

Ionic liquids are compounds with very different rheological parameters. Depending on many factors, such as (i) the length of the alkylated chain used or (ii) the type of anion, they can be compounds of very low viscosity as well as those with a viscosity resembling glue [47]. In fact, in some application areas, it is necessary to introduce additional substances that are designed to adjust the ionic liquids to a specific application, not only by changing the rheological properties, but sometimes also by additional interactions with ILs [48].

Ethylene glycol was not accidentally used in this work, which was supposed to fulfill the following tasks: (i) to reduce the viscosity of long-chain ionic liquids and at the same time increase their compatibility in combination with the epoxy resin molecule, (ii) due to its emulsifying properties [36,37], to form stable curing mixtures, (iii) be a substance capable of absorbing ammonia [49] and formaldehyde or eventually carbon dioxide [50] that could be released from the IL over time of its storage and (iv) remain in the structure of the cured resin, thanks to its high boiling point exceeding the temperature of the cross-linking process. In addition, during the study, the effect of ethylene glycol on slowing down the pH change process was observed, which was systematically controlled before each application experiment, using simple strip tests. Moreover, no ammonia odor was observed in mixture samples stored for 60 days. We suppose that through emulsification and connection with the structure of the IL, the EG undergoes a specific incorporation into the structure of the cation through a network of hydrogen bonds. Thus, this stabilizes the quaternary structure of HMTA and slows down the process of its breakdown. However, this is only an assumption requiring much extended research and theoretical calculations that would not affect the application presented in the work.

### 3.3. Curing Process and the Mechanism

**Figure 4:**
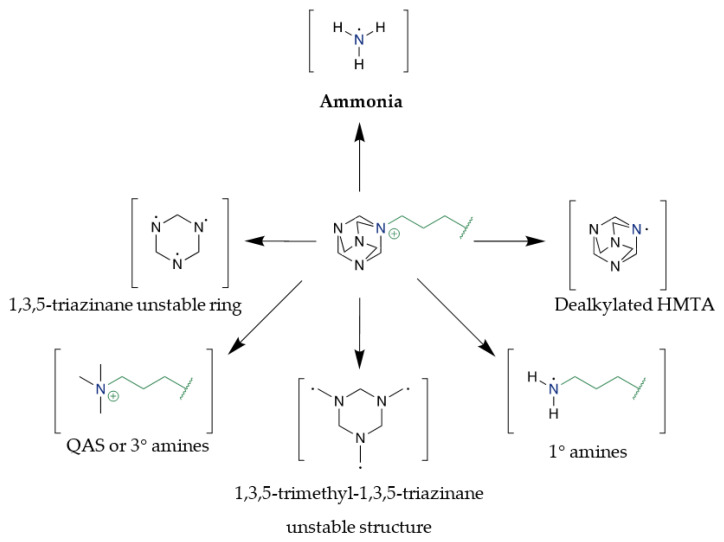
Proposed pathways of thermal decomposition of HMTA-based ILs.

The aim of this work was to demonstrate the use of new ionic liquids as active ingredients in the curing of epoxy resins. For this purpose, studies of the curing process were carried out, drawing key conclusions: (i) HMTA-based ionic liquids can successfully initiate the curing of epoxies, (ii) the low stability of these compounds favors their use in this area, resulting in lower temperatures of the curing process, (iii) the choice of anion affects the curing process itself, in particular the temperature range of the process. The quaternary hexamethylenetetramine cation can disintegrate under the influence of temperature into a whole spectrum of different types of molecules. Most of them are highly reactive, energetically unstable molecules, and able to react quickly with the epoxide groups of the resin. In Figure 4 several potential transition structures that may be generated during the process of thermal decomposition of HMTA-ILs have been proposed. The presented structures were inspired by the Delepine reaction mechanism, but also by data on HMTA decay directions [40,41]. The process generates, among others, primary amines based on an alkyl chain that was attached to the generic cation structure. In addition, the process may also produce ammonia, which is easily reacted with the epoxy group to form a quaternary adduct, which is unstable and quickly initiates a chain reaction leading to full cross-linking of the resin structure. 

Undoubtedly, in the case of curing the resin with ionic liquids based on HMTA, we are dealing with independently occurring processes of disintegration and simultaneous reaction with the epoxy groups present in the DGEBA molecule. In addition, the epoxy group can react with both basic (amines) and acidic compounds, which means that the anions present in the ionic liquid also react with the resin, leading to the formation of fully cross-linked structures. This phenomenon can be defined as cationic or anionic polymerization depending on which part of the ionic liquid is reacting with the epoxide at a given moment [21]. Both processes usually take place simultaneously, but if they occur at different temperatures, they are sometimes observable as separate polymerization peaks in thermal analysis. From the considerations regarding the ability to initiate the epoxy resin curing process, (i) due to their high thermal stability, and (ii) the lack of ionic liquid characteristics that would allow these compounds to be classified into this group, halide derivatives were excluded. So, the curing process of epoxy resin initiated by systems based on ionic liquids with TFSI anion is a process independent of the structure of the cation and its chain length. For all the tested compounds, the polymerization start, peak and end temperatures were similar, with the only exception being the derivative with an ethyl chain, which, probably due to its short chain, quickly joins the curing process and does not require the time necessary to generate appropriate, active transitional structures. What is more, this thesis becomes more likely with the information that a much greater amount of energy is released in the thermal process than in the case of analogous structures with longer carbon chains. In general, it can be concluded that the initiators based on HMTA–TFSI-ILs are characterized by a predictable and independent curing process. On the other hand, the polymerization reaction initiated by dicyanamides is characterized by a single thermal peak with a maximum temperature peak that depends on the chain length and generally decreases as it increases. More precisely, we assume that with the increase in the length of the alkyl chain in the cation structure, the thermal stability of the compounds decreases, which means that active molecules directly participating in the reaction with epoxides are generated faster. At the same time, we noticed differences in the polymerization start temperature depending on whether the alkyl chain has an even or odd number of carbon atoms. We suggest that the reason for this phenomenon may be that structures with an odd number of carbon atoms in the chain will decay faster, especially at the very beginning of the process, through an energetically lower favorable structure. Against the background of all the tested substances, the **5c** derivative stands out the most, for which the polymerization start temperature is significantly lower than for the rest of the tested substances. However, the temperature peak for this substance is slightly lower than for the other representatives of this group, and the polymerization end temperature remains the same for all. We suggest that the reason for this may be the fact that the hexyl alkyl chain strains the cation structure significantly and the effect of ethylene glycol is not able to compensate for this phenomenon, so the molecule undergoes a slight disintegration, generating active initiators of resin polymerization, especially at the beginning after supplying temperature to the system, which can be seen by the recorded low polymerization start temperature and high process energy. 

### 3.4. Hardness of the Materials

For additional characterization of the cured materials obtained, a simple measurement of the hardness of the resin after curing was chosen. Such a parameter allows one to quickly determine the effect of the tested substances on the final product and define certain regularities. The results showed that with the increase in the length of the carbon chain in the cation structure, the brittleness of the obtained material increases, which in some cases made it impossible to measure the hardness. We suggest that such a hardness test result correlates with the values of water absorbed by ionic liquids, and thus with the level of hygroscopicity of a particular ionic liquid. Moreover, as a rule, ionic liquids with TFSI anions are hydrophobic in nature, while dicyanamides are representatives of mainly hydrophilic compounds and it is for this group of compounds that information about significant brittleness has been reported more often. In addition, the brittle effect of the structure can sometimes also be caused by the presence of ethylene glycol.

In conclusion, it was proved during research that by using the first stage of the Delepine reaction, leading to the production of hexamethylenetetramine quaternary ammonium salts, it was possible to obtain a series of new ionic liquids that can be successfully used in epoxy resin curing mixtures operating only at elevated temperatures. What is more, the use of ethylene glycol as a component of such mixtures is presented, which simultaneously performs many functions important for the proposed area of application as well as for the obtained ionic liquids, especially in terms of their stability.

## 4. Materials and Methods

### 4.1. Materials

All reagents were purchased from suppliers: (i) Merck (alkylating agents, hexamethylenetetramine, triethylenetetramine, isophorone diamine, lithium bis(trifluoromethylsulfonyl)imide, sodium dicyanamide, ethylene glycol, organic solvents, reagents necessary to perform analyzes), and (ii) abcr GmbH (bisphenol A diglycidyl ether, DGEBA, silver nitrate). The minimum purity for alkylating agents, amines (beyond triethylenetetramine—97%) and compounds used for metathesis is 99%, while all solvents had a minimum HPLC grade. DGEBA was 85% purity.

### 4.2. Synthesis

#### 4.2.1. Bromides

To obtain products **1a**–**5a**, a solution of hexamethylenetetramine (1.0 eq.) in chloroform was placed in a two-necked round-bottomed flask equipped with a reflux condenser with simultaneous stirring on magnetic stirrer. Then, corresponding alkyl bromide (1.0 eq.) was added dropwise at room temperature. After that, a white precipitate was observed immediately. After the time required to complete the reaction (controlled with IC analysis), a large amount of white precipitate was filtered through a sintered funnel, then washed with hexane (3 × 10.0 mL) and diethyl ether (3 × 10.0 mL). Products were dried under vacuum (24 h) and recrystallized from a mixture of methanol and diethyl ether to furnish pure compounds.

Compound **1a**

Product form: white solid; yield: 99.6%; water content: 0.47 wt%; IC: t_R_ = 4.67 min (cation), 4.14 min (anion), Purity: 99.6%; ^1^H NMR (400 MHz, D_2_O): δ = 5.05 (s, 6H), 4.69 (d, *J* = 12.4 Hz, 3H), 4.54 (d, *J* = 12.5 Hz, 3H), 2.98 (q, *J* = 7.5 Hz, 2H), 1.25 (t, *J* = 7.4 Hz, 3H); ^13^C {^1^H} NMR (100 MHz, D_2_O): δ = 77.72, 70.14, 52.66, 4.95; MS (ESI): *m/z* (%) = 169 ([M]^+^, 100), 155 ([HMTA-CH_2_]^+^), 140 ([HMTA]^+^).

Compound **2a**

Product form: white solid; yield: 99.0%; water content: 0.17 wt%; IC: t_R_ = 4.89 min (cation), 4.17 min (anion), Purity: 99.0%; ^1^H NMR (400 MHz, D_2_O): δ = 5.04 (s, 6H), 4.63 (d, *J* = 14.9 Hz, 3H), 4.52 (d, *J* = 13.4 Hz, 3H), 2.81 (m, 2H), 1.67 (ddd, *J* = 15.3, 7.4, 5.3 Hz, 2H), 0.89 (t, *J* = 7.3 Hz, 3H); ^13^C {^1^H} NMR (100 MHz, D_2_O): 77.65, 70.15, 58.32, 20.77, 13.63; MS (ESI): *m/z* (%) = 183 ([M]^+^, 100), 155 ([HMTA-CH_2_]^+^), 140 ([HMTA]^+^).

Compound **3a**

Product form: white solid; yield: 99.1%; water content: 0.68 wt%; IC: t_R_ = 5.12 min (cation), 4.16 min (anion), Purity: 99.1%; ^1^H NMR (400 MHz, D_2_O): δ = 5.06 (s, 6H), 4.65 (d, *J* = 15.5 Hz, 3H), 4.54 (d, *J* = 13.0 HZ, 3H), 2.87 (m, 2H), 1.65 (ddd, *J* = 15.6, 12.6, 7.5 Hz, 2H), 1.32 (dd, *J* = 14.8, 7.4 Hz, 2H), 0.89 (t, *J* = 7.4 Hz, 3H); ^13^C {^1^H} NMR (100 MHz, D_2_O): 78.13, 70.14, 60.45, 26.82, 19.61, 14.03; MS (ESI): *m/z* (%) = 197 ([M]^+^, 100), 168 ([HMTA-CH_2_-CH_2_]^+^, 155 ([HMTA-CH_2_]^+^), 140 ([HMTA]^+^).

Compound **4a**

Product form: white solid; yield: 99.1%; water content: 0.57 wt%; IC: t_R_ = 5.78 min (cation), 4.12 min (anion), Purity: 99.1%; ^1^H NMR (400 MHz, D_2_O): δ = 5.05 (s, 6H), 4.66 (d, *J* = 13.5 Hz, 3H), 4.55 (d, *J* = 13.1 Hz, 3H), 2.85 (m, 2H), 1.65 (dd, *J* = 15.4, 7.5 Hz, 2H), 1.31 (m, 4H), 0.83 (t, *J* = 7.1 Hz, 3H); ^13^C {^1^H} NMR (100 MHz, D_2_O): 77.52, 70.93, 60.43, 31.57, 25.32, 19.70, 13.05; MS (ESI): *m/z* (%) = 211 ([M]^+^, 100), 182 ([HMTA-CH_2_-CH_2_-CH_2_]^+^, 168 ([HMTA-CH_2_-CH_2_]^+^, 155 ([HMTA-CH_2_]^+^), 140 ([HMTA]^+^).

Compound **5a**

Product form: white solid; yield: 99.0%; water content: 0.42 wt%; IC: t_R_ = 6.34 min (cation), 4.10 min (anion), Purity: 99.1%; ^1^H NMR (400 MHz, D_2_O): δ = 5.03 (s, 6H), 4.67 (d, *J* = 13.1 Hz, 3H), 4.51 (d, *J* = 12.7 Hz, 3H), 2.84 (dd, *J* = 11.9, 5.5 Hz, 2H), 1.63 (dd, *J* = 15.8, 7.8 Hz, 2H), 1.26 (m, 6H), 0.80 (t, *J* = 6.9 Hz, 3H); ^13^C {^1^H} NMR (100 MHz, D_2_O): 78.12, 70.12, 57.32, 30.25, 25.47, 21.64, 19.10, 13.15; MS (ESI): *m/z* (%) = 225 ([M]^+^, 100), 182 ([HMTA-CH_2_-CH_2_-CH_2_]^+^, 155 ([HMTA-CH_2_]^+^), 140 ([HMTA]^+^).

#### 4.2.2. Bis(trifluoromethane)sulfonimides

Compounds **1b**–**5b** were obtained according to a slightly modified, previously reported procedure. Corresponding bromide (**1a**–**5a**, 1.0 eq.) was dissolved in deionized water (100.0 mL) and transferred into a separating funnel. Then, methylene chloride was added followed by an aqueous solution of lithium bis(trifluoromethylsulfonyl)imide (80%, 1.1 eq.). The obtained mixture was vigorously shook for a few minutes and then the separated organic layer was washed with deionized water (3 × 20.0 mL) and then deionized water with small portion of lithium bis(trifluoromethylsulfonyl)imide (80%, 0.1 eq., 1 × 20.0 mL). The washing procedure was finished in an absence of bromide anion in the water layer (silver nitrate test, and washed until no observable yellow silver bromide precipitation). To obtain pure product (**1b**–**5b**), methylene chloride from the organic layer was evaporated under reduced pressure and bis(trifluoromethylsulfonyl)imide ionic liquid was dried under vacuum (24 h).

Compound **1b**

Product form: slightly yellow liquid; yield: 98.8%; water content: 0.31 wt%; IC: t_R_ = 4.57 min (cation), 18.58 min (anion), Purity: 99.8%.

Compound **2b**

Product form: yellow liquid; yield: 97.7 %; water content: 0.23 wt%; IC: t_R_ = 4.94 min (cation), 18.71 min (anion), Purity: 99.6%.

Compound **3b**

Product form: transparent liquid; yield: 99.1%; water content: 0.28 wt%; IC: t_R_ = 5.19 min (cation), 18.73 min (anion), Purity: 99.5%.

Compound **4b**

Product form: yellow liquid; yield: 98.9%; water content: 0.49 wt%; IC: t_R_ = 5.81 min (cation), 18.69 min (anion), Purity: 99.1%

Compound **5b**

Product form: yellow, viscous liquid; yield: 97.6%; water content: 0.38 wt%; IC: t_R_ = 6.31 min (cation), 18.71 min (anion), Purity: 99.1%.

#### 4.2.3. Dicyanamides

Compounds **1c–5c** were obtained according to a slightly modified previous reported procedure. A precisely weighed amount of corresponding bromide (**1a**–**5a**, 1.0 eq.) was placed in the flask and dissolved in deionized water (30.0 mL). Then, freshly prepared silver dicyanamide was added (1.0 eq.) and the mixture was stirred on a magnetic stirrer, protected from a sunlight. The exchange process was monitored by ion chromatography. After completion of the reaction, the yellow precipitate of silver bromide was separated on a sintered funnel using a glass fiber filter, and washed with water (3 × 15.0 mL). The filtrate was evaporated in a vacuum evaporator, and then the obtained compound was purified by dissolution in anhydrous methylene chloride and repeated filtration. To obtain pure product (**1c**–**5c**), methylene chloride was evaporated under reduced pressure and dicyanamide ionic liquid was dried under vacuum (24 h).

Compound **1c**

Product form: yellow solid; yield: 96.2%; water content: 0.73 wt%; IC: t_R_ = 4.59 min (cation), 6.66 min (anion), Purity: 98.1%.

Compound **2c**

Product form: slightly yellow solid; yield: 87.9%; water content: 0.43 wt%; IC: t_R_ = 4.92 min (cation), 6.59 min (anion), Purity: 97.9%.

Compound **3c**

Product form: yellow solid; yield: 97.5%; water content: 0.86 wt%; IC: t_R_ = 5.16 min (cation), 6.58 min (anion), Purity: 98.1%.

Compound **4c**

Product form: yellow, viscous solid; yield: 93.9%; water content: 0.69 wt%; IC: t_R_ = 5.83 min (cation), 6.63 min (anion), Purity: 97.8%.

Compound **5c**

Product form: yellow, viscous solid; yield: 97.0%; water content: 0.33 wt%; IC: t_R_ = 6.29 min (cation), 6.60 min (anion), Purity: 97.5%.

### 4.3. Ion Chromatography

All experiments were conducted with the use of ion chromatography, both regarding the control of the reaction course and the determination of the purity of the obtained compounds, which were performed using Eco-IC apparatus (Metrohm, Herisau, Switzerland) in accordance with the procedures published in previous works [12,21].

### 4.4. Thermal Analysis

#### 4.4.1. Differential Scanning Calorimetry (DSC) for ILs

Melting point/glass transition/decomposition temperature analyses were determined using the DSC 3 apparatus (Mettler Toledo, Greifensee, Switzerland), according to the method reported in previous work [12,21] and described in Supplementary Materials.

#### 4.4.2. Differential Scanning Calorimetry (DSC) for Epoxy Systems

The cross-linking process of the epoxy resin was tested using a DSC 8500 apparatus (PerkinElmer, Waltham, MA, USA), according to a slightly modified method described in the previous work [21] and described in Supplementary Materials.

### 4.5. Water Content

Determination of the water content in the obtained compounds was carried out using the Karl Fischer titration apparatus (Metrohm, Herisau, Switzerland) in accordance with the method described in previous works [12,21].

### 4.6. NMR Analysis

NMR spectra were recorded using a 400 MHz (^1^H) and 100 MHz (^13^C) Ascend spectrometer (Bruker, Billerica, MA, USA) in commercially available D_2_O solvent. The value of standard measurement uncertainty did not exceed ±0.02 ppm.

### 4.7. ESI-MS Analysis

ESI-MS analysis were conducted using a HPLC/MS apparatus (Waters Corporation, Milford, MA, USA), according to the procedure described in the previous work [12].

### 4.8. Curing Process

All experiments regarding the curing process were performed in accordance with the procedure described in the previous work [21]. For microscopic documentation of cured samples see the Supplementary Materials.

### 4.9. Hardness Measurments

All experiments regarding the hardness measurements were performed in accordance with the procedure described in the previous work [21].

## Figures and Tables

**Table 1:** Parameters of the synthesis of HMTA derivatives.

Compound	Yield [%]	Purity [%]	Water Content [wt%]
**1a**	99.6	99.6	0.47
**2a**	99.0	99.0	0.17
**3a**	99.1	99.1	0.68
**4a**	99.1	99.1	0.57
**5a**	99.0	99.1	0.42

**Table 2:** Thermal properties of the HMTA-based compounds obtained in this research.

Compound	T_m_ [°C]	T_g_ [°C]	T_decomp_ [°C]
**1a**	-	-	168.3 ^a^
**2a**	-	-	146.5 ^a^
**3a**	-	-	155.4 ^a^
**4a**	-	-	143.6 ^a^
**5a**	-	-	152.4 ^a^
**1b**	-	−5.0	119.3 ^b^
**2b**	-	−16.6	126.8 ^b^
**3b**	-	−17.1	126.3 ^b^
**4b**	-	−29.1	121.6 ^b^
**5b**	-	−19.9	134.7 ^b^
**1c**	69.1	-	120.3 ^b^
**2c**	57.4	-	135.3 ^b^
**3c**	72.1	-	101.6 ^b^
**4c**	75.5	-	124.0 ^b^
**5c**	86.6	-	126.8 ^b^

^a^ Decomposition onset peak, ^b^ Start of exothermic decomposition during DSC measurement.

**Table 3:** Thermal properties of the curing process.

System	Anion	T_onset_ [°C]	T_max_ [°C]	T_end_ [°C]	ΔH [J/g]
**1b**/EG/DGEBA	TFSI	109.4	137.0	164.8	84.4
		200.7	223.5	236.9	24.3
**2b**/EG/DGEBA		122.2	148.1	201.5	26.5
		215.9	224.4	236.4	11.0
**3b**/EG/DGEBA		122.1	154.4	185.6	19.8
		210.3	218.9	239.5	11.7
**4b**/EG/DGEBA		119.1	147.0	205.7	29.3
		221.0	229.5	242.3	8.9
**5b**/EG/DGEBA		119.8	149.0	193.2	28.5
		209.3	221.7	223.0	7.2
**1c**/EG/DGEBA	DCA	140.8	170.7	224.0	49.5
**2c**/EG/DGEBA		123.6	164.7	213.2	81.6
**3c**/EG/DGEBA		140.0	163.9	188.1	49.8
**4c**/EG/DGEBA		129.5	165.1	199.1	23.0
**5c**/EG/DGEBA		101.3	151.8	210.6	151.5

**Table 4:** Hardness measurements results.

Compound	Hardness [D]
RM1/RM2	74/71
**1b**	78
**2b**	80
**3b**	76
**4b**	81
**5b**	n/a
**1c**	87
**2c**	n/a
**3c**	89
**4c**	n/a
**5c**	80

n/a—not available due to too high brittleness of the samples.

## Data Availability

Not applicable.

## Supplementary Materials

The following supporting information can be downloaded at: https://www.mdpi.com/article/10.3390/molecules28020892/s1, 1. NMR spectra; 2. DSC thermograms for ILs measurements; 3. DSC thermograms for curing process; 4. Microscopic pictures of cured samples; Figure S1. Microscopic pictures of cured samples.

## Sample Availability

Not available.
