# Supplementary material for: Synthesis and Behavior of Hexamethylenetetramine-Based Ionic Liquids as an Active Ingredient in Latent Curing Formulations with Ethylene Glycol for DGEBA
Source: Molecules. 2023 Jan 16;28(2):892. doi: 10.3390/molecules28020892 (PMC9863291; doi:10.3390/molecules28020892)
Supplement: Supplementary file 1 [file molecules-28-00892-s001.zip › molecules-2100039-supplementary.pdf]

Synthesis and behavior of Hexamethylenetetramine-based ionic liquids as an active ingredient in latent curing formulations with ethylene glycol for DGEBA

Dawid Zielinski [1,2]*, Andrea Szpecht [1], Paulina Hinc [2] and Marcin Smiglak [1]

[1] Poznan Science and Technology Park, Adam Mickiewicz University Foundation, ul. Rubiez 46, 61-612 Poznań, Poland
[2] Faculty of Chemistry, Adam Mickiewicz University in Poznań, ul. Uniwersytetu Poznańskiego 8, 61-614 Poznań, Poland
* Correspondence: dawid.zielinski@ppnt.poznan.pl

Supporting Information

Content

1. *NMR spectra*
   1.1. Compound **1a** 2
   1.2. Compound **2a** 3
   1.3. Compound **3a** 4
   1.4. Compound **4a** 5
   1.5. Compound **5a** 6
2. *DSC thermograms for ILs measurements*
   2.1. Procedure and parameters 7
   2.2. Compound **1a** 7
   2.3. Compound **2a** 7
   2.4. Compound **3a** 8
   2.5. Compound **4a** 8
   2.6. Compound **5a** 8
   2.7. Compound **1b** 9
   2.8. Compound **2b** 9
   2.9. Compound **3b** 9
   2.10. Compound **4b** 10
   2.11. Compound **5b** 10
   2.12. Compound **1c** 10
   2.13. Compound **2c** 11
   2.14. Compound **3c** 11
   2.15. Compound **4c** 11
   2.16. Compound **5c** 12
3. *DSC thermograms for curing process*
   3.1. Procedure and parameters 13
   3.2. Compound **1b** 13
   3.3. Compound **2b** 14
   3.4. Compound **3b** 14
   3.5. Compound **4b** 15
   3.6. Compound **5b** 15
   3.7. Compound **1c** 16
   3.8. Compound **2c** 16
   3.9. Compound **3c** 17
   3.10. Compound **4c** 17
   3.11. Compound **5c** 18
4. *Microscopic pictures of cured samples* 19
5. *References* 21

1. *NMR spectra*

1.1. Compound **1a**

^{1}H NMR

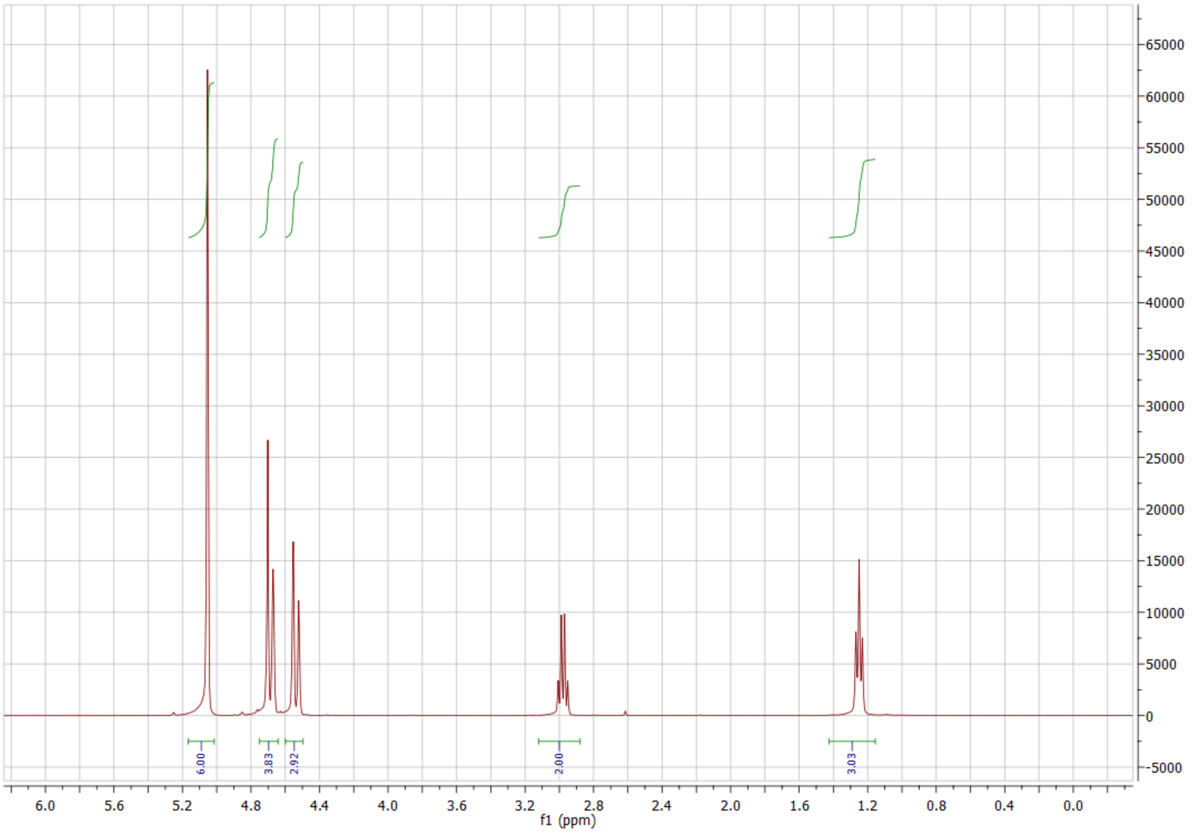

^{13}C NMR

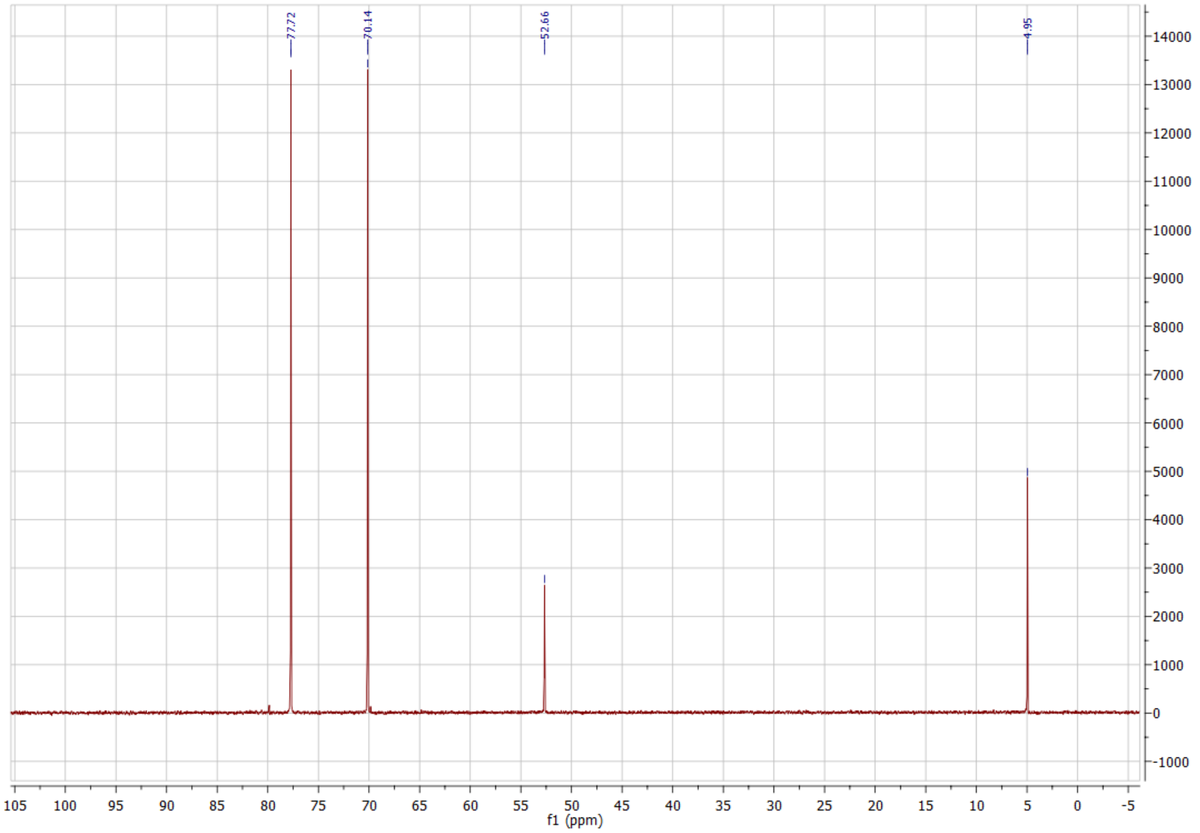

1.2. Compound **2a**

^1H NMR

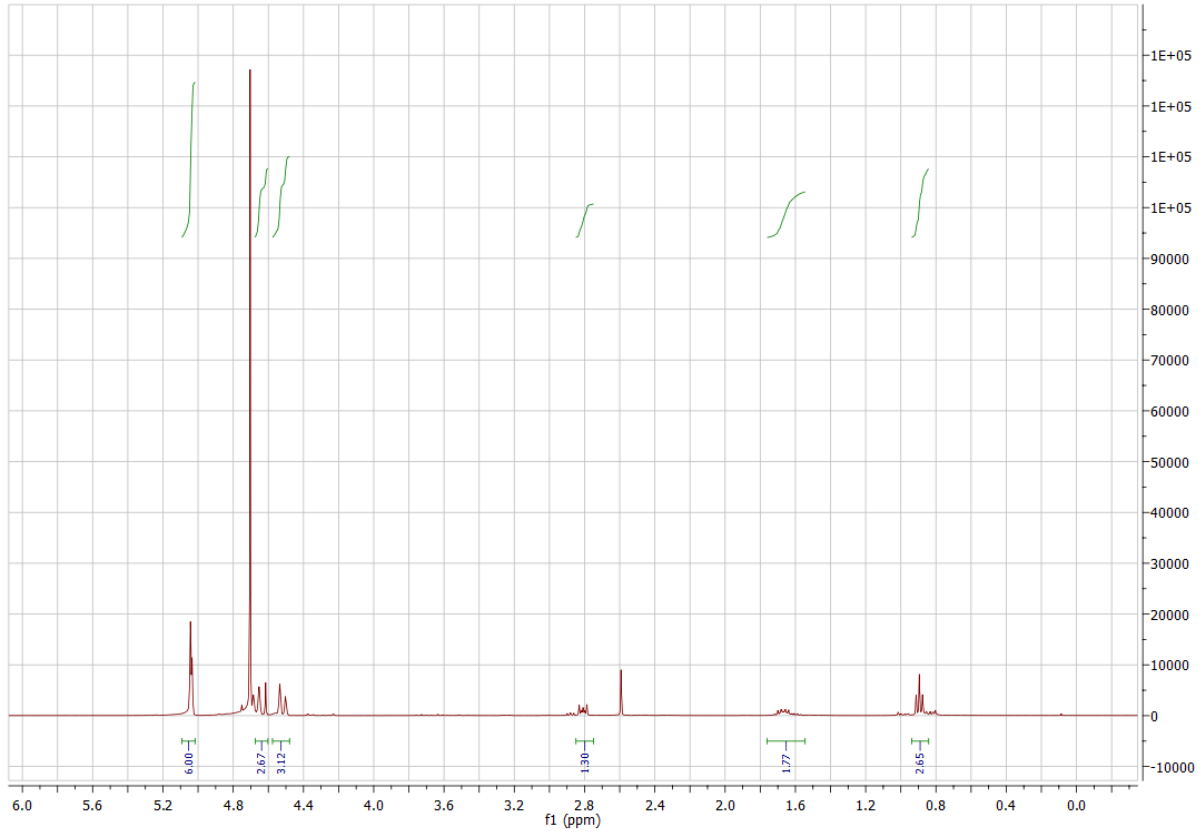

^{13}C NMR

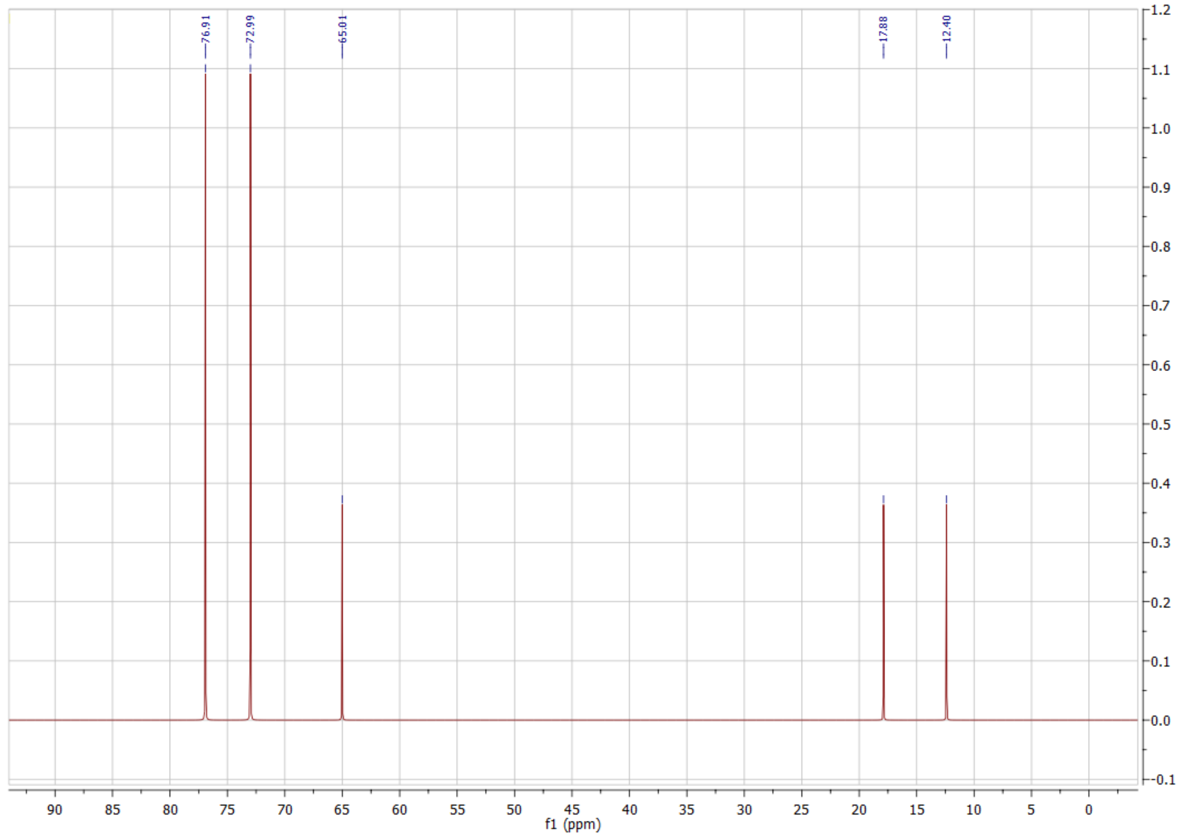

1.3. Compound **3a**

^{1}H NMR

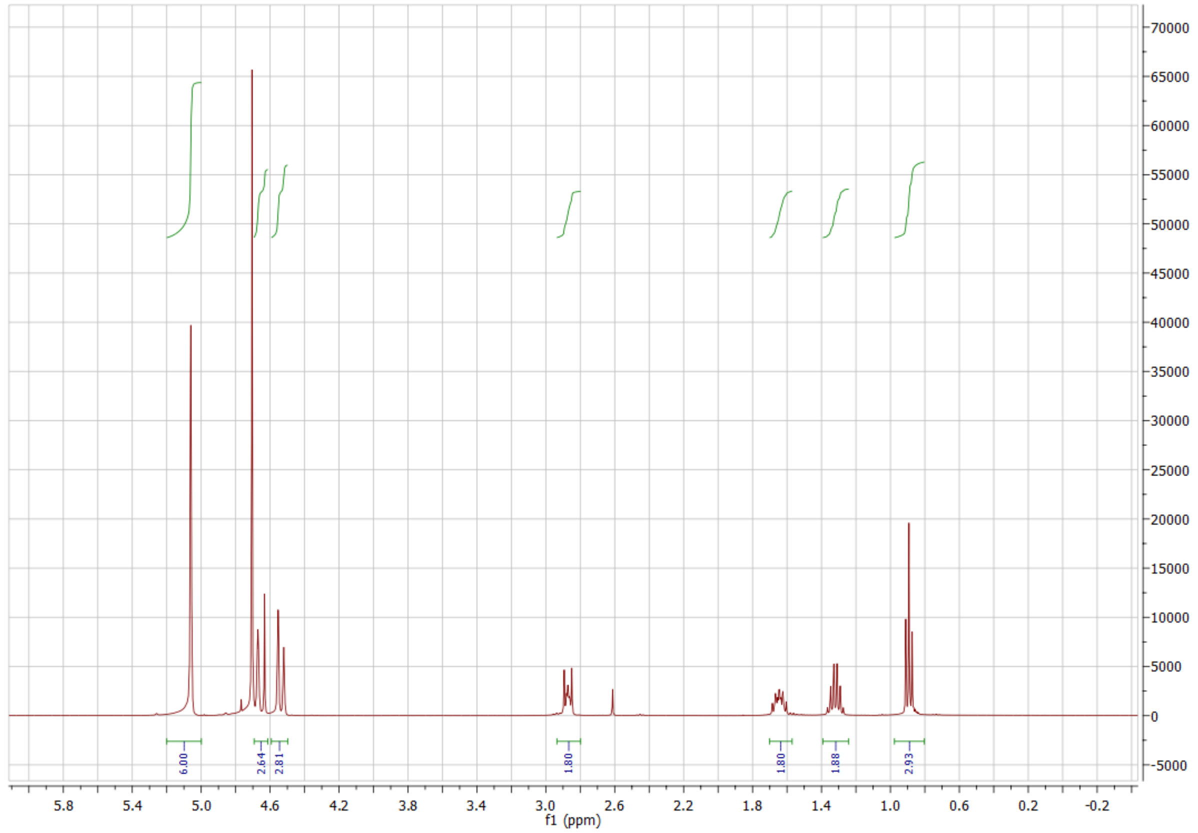

^{13}C NMR

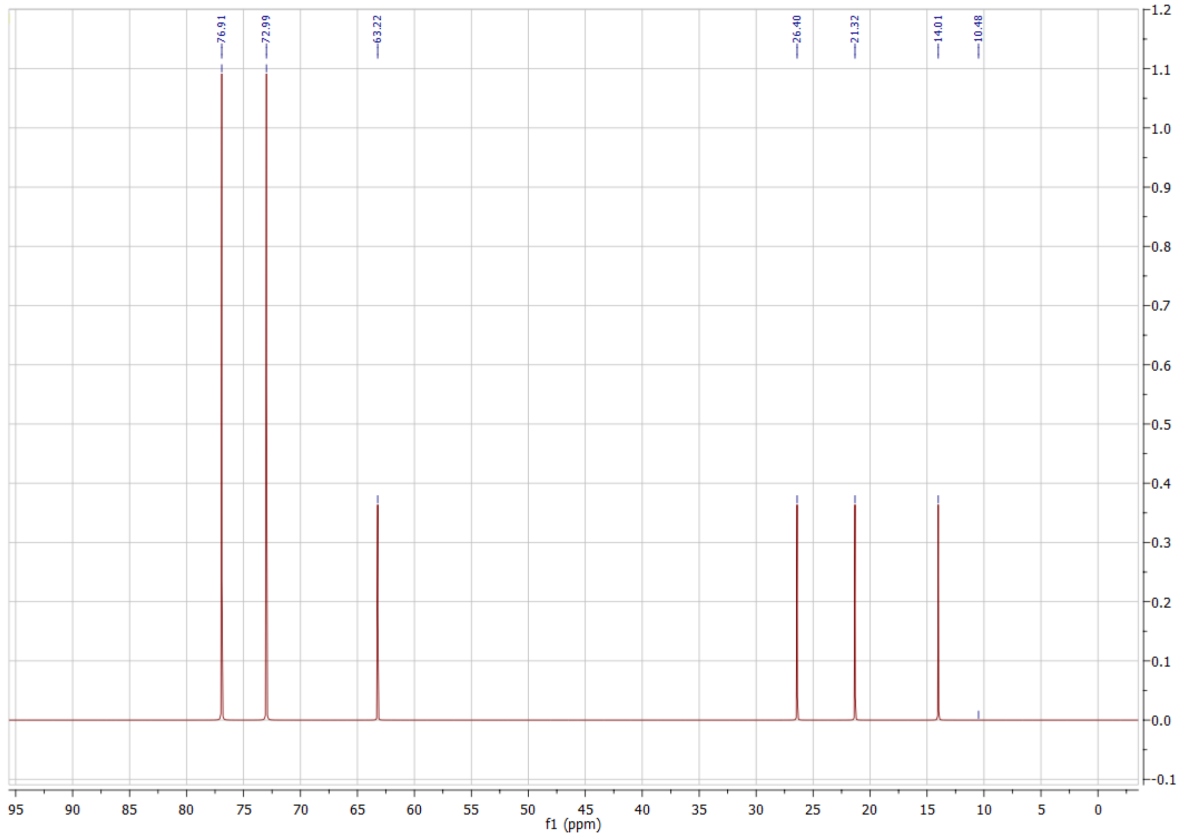

1.4. Compound **4a**

^{1}H NMR

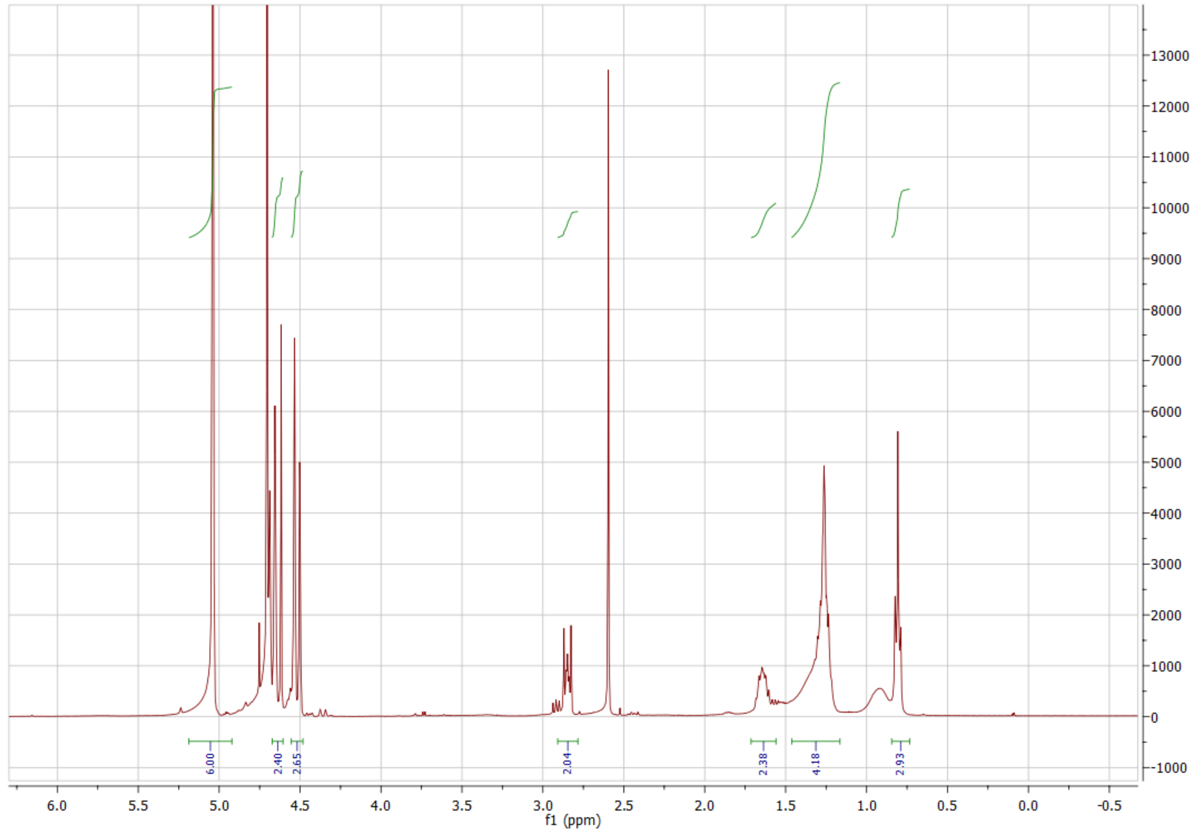

^{13}C NMR

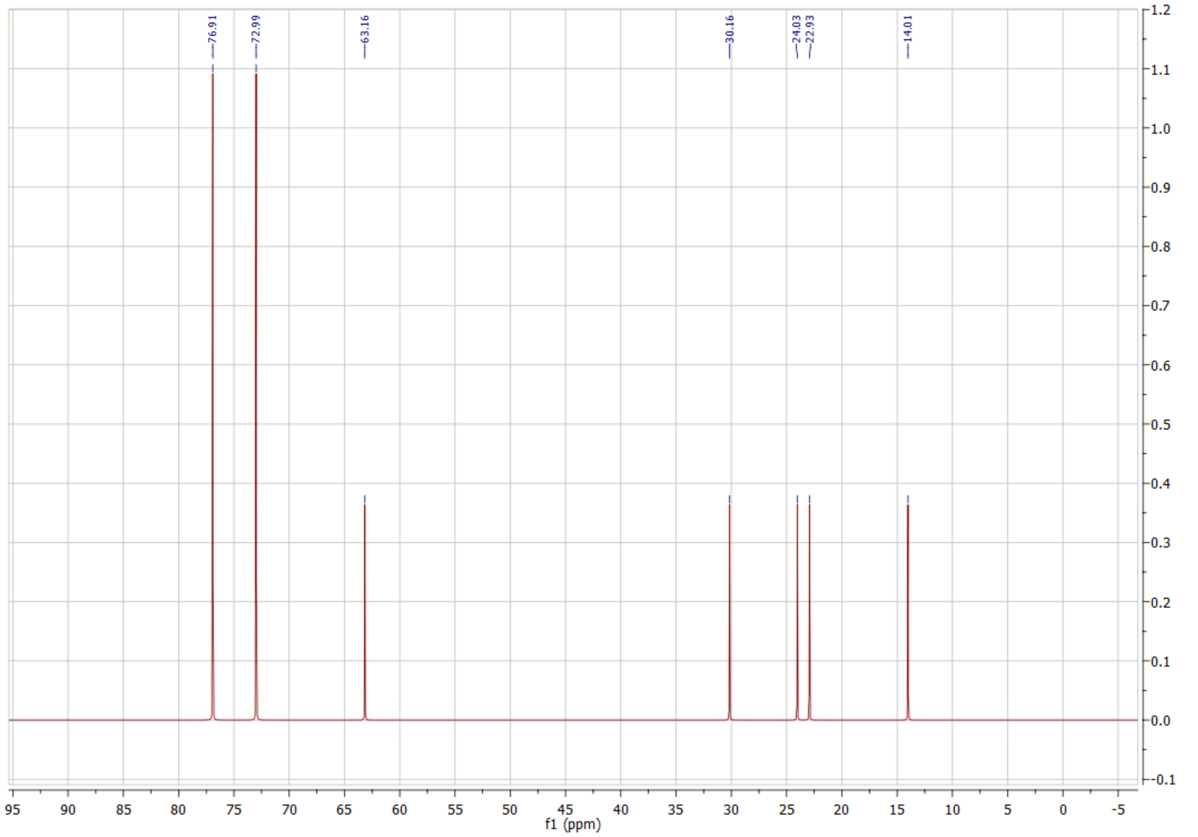

1.5. Compound **5a**

^{1}H NMR

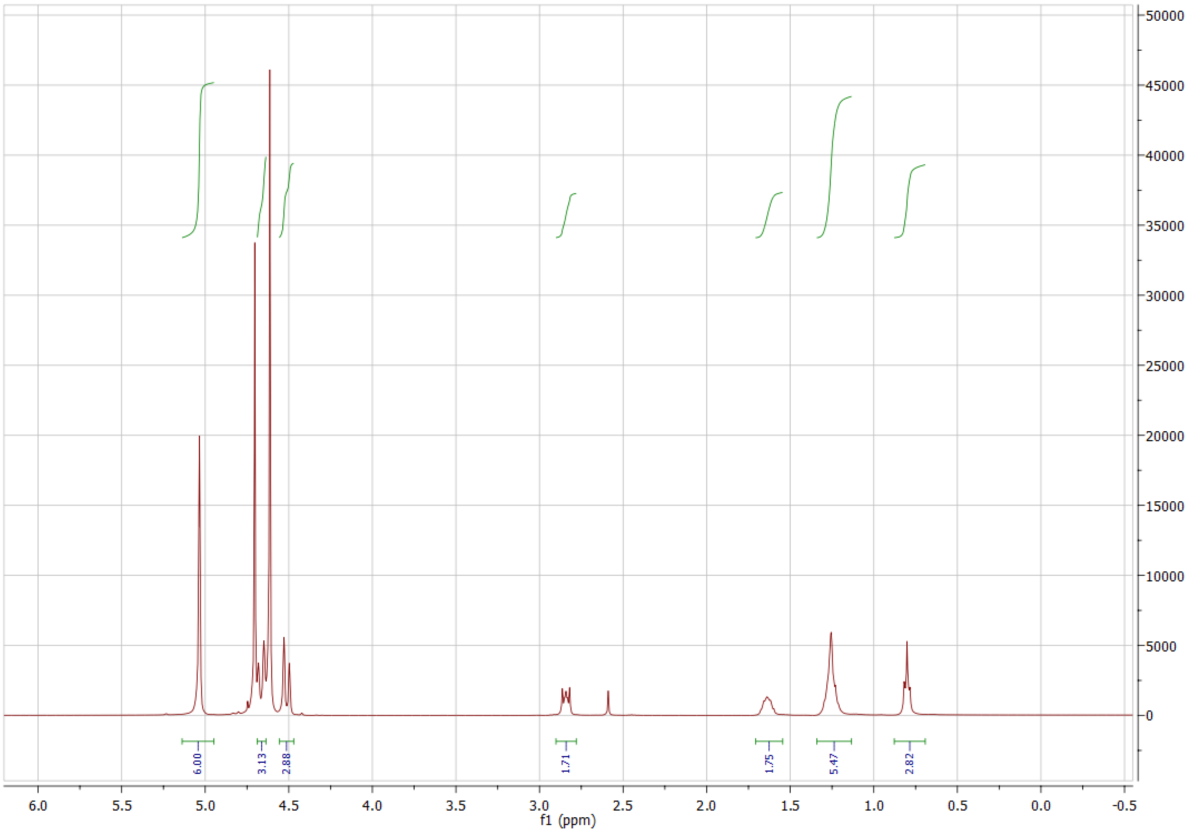

^{13}C NMR

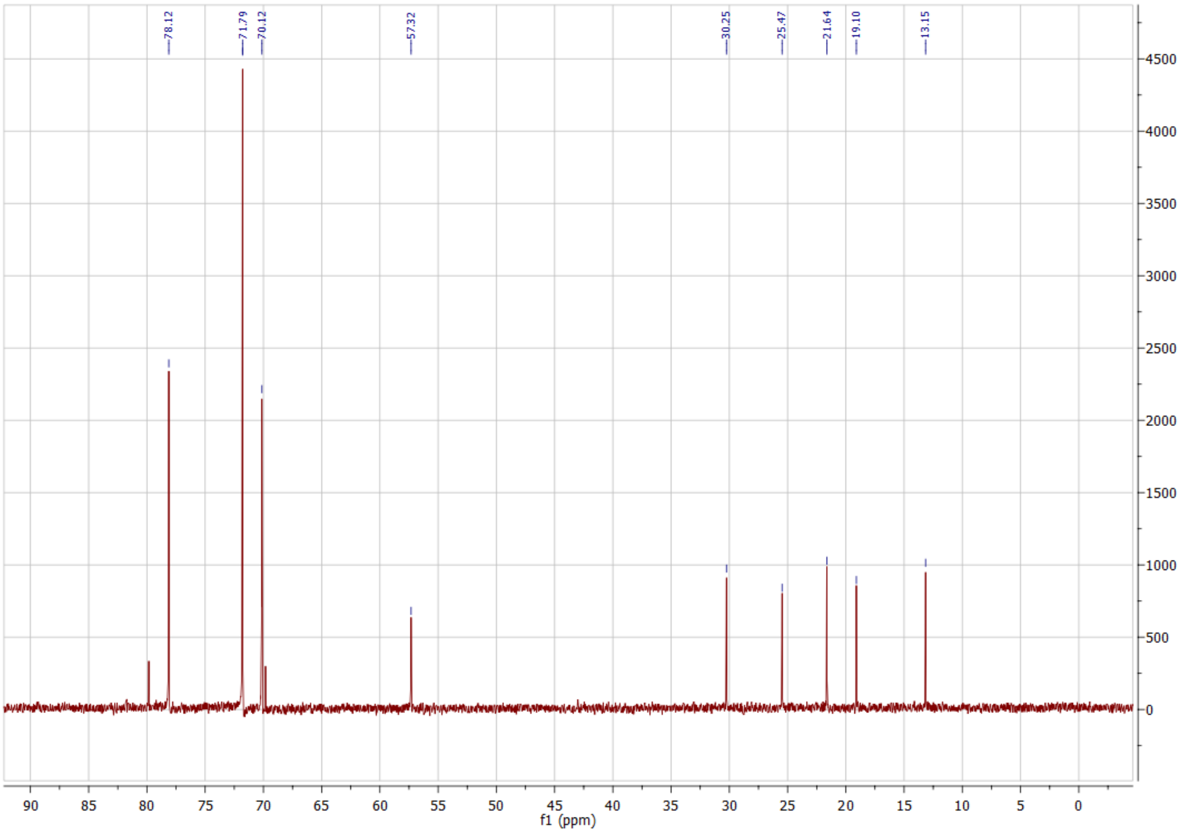

2. *DSC thermograms for ILs measurements*

2.1. Procedure and parameters

Melting point/glass transition analyses were determined by running DSC experiments with use of differential scanning calorimeter, cooled with an immersion cooler. The calorimeter was calibrated for temperature and cell constants using high purity indium (melting temperature: 156.7 °C; specific enthalpy of melting: 28.71 J·g^{-1}) and zinc (melting temperature: 419.6 °C; specific enthalpy of melting: −107.5 J·g^{-1}). Data were collected at atmospheric pressure. The samples were initially cooled from room temperature, at a rate of 10 °C·min^{-1}, to −80 °C. At this temperature, they were held for 10 min isotherm, prior to two cycles of heating and cooling at rates of 10 °C·min−1 spaced by 10 min isothermal holding at the lower (−80 °C) and upper (130/200 °C) endpoint temperatures. The upper endpoint temperature was set to 130 °C in the first cycle and 200 °C for the second cycle. For all experiments, samples in the weight range between 10 and 15 mg were used in aluminium sample pans, sealed with lids with a pin hole. An empty sample pan served as the reference. The temperatures reported for the glass transition and melting were established as the peak temperatures, for the endothermic changes in heat flow. The temperatures reported for the decomposition temperatures visible on DSC chart were onset temperatures [1].

2.2. Compound **1a**

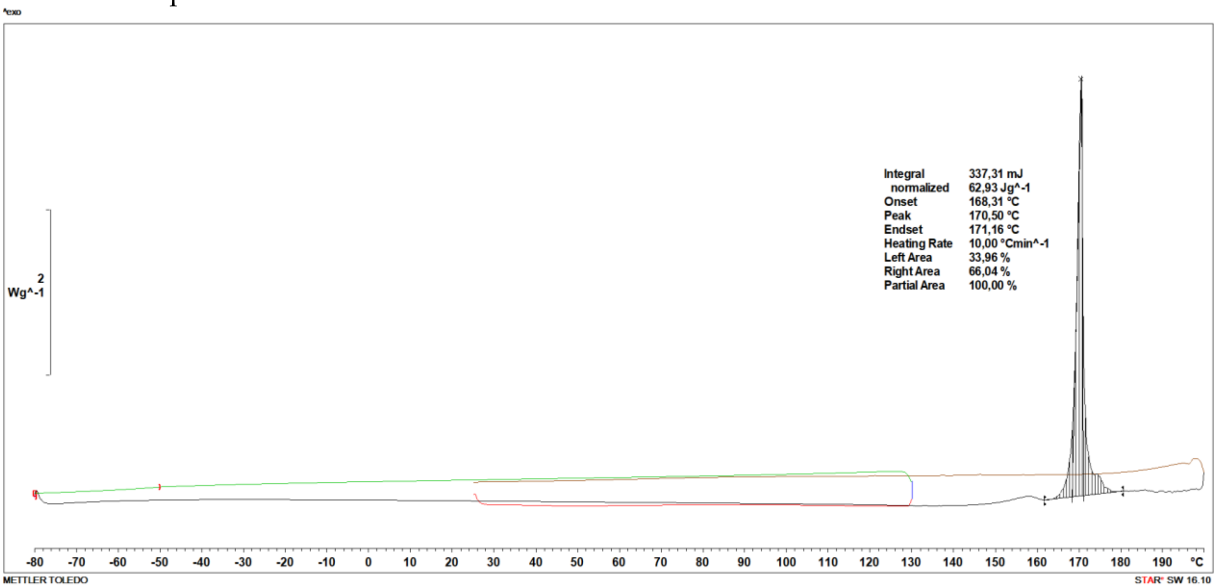

2.3. Compound **2a**

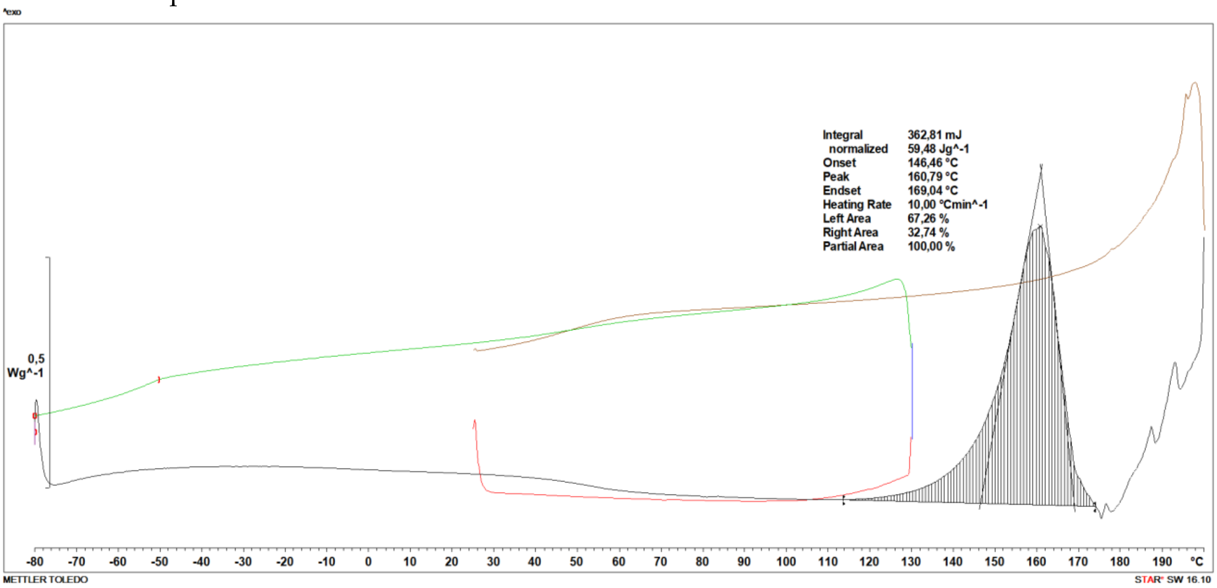

2.4. Compound **3a**

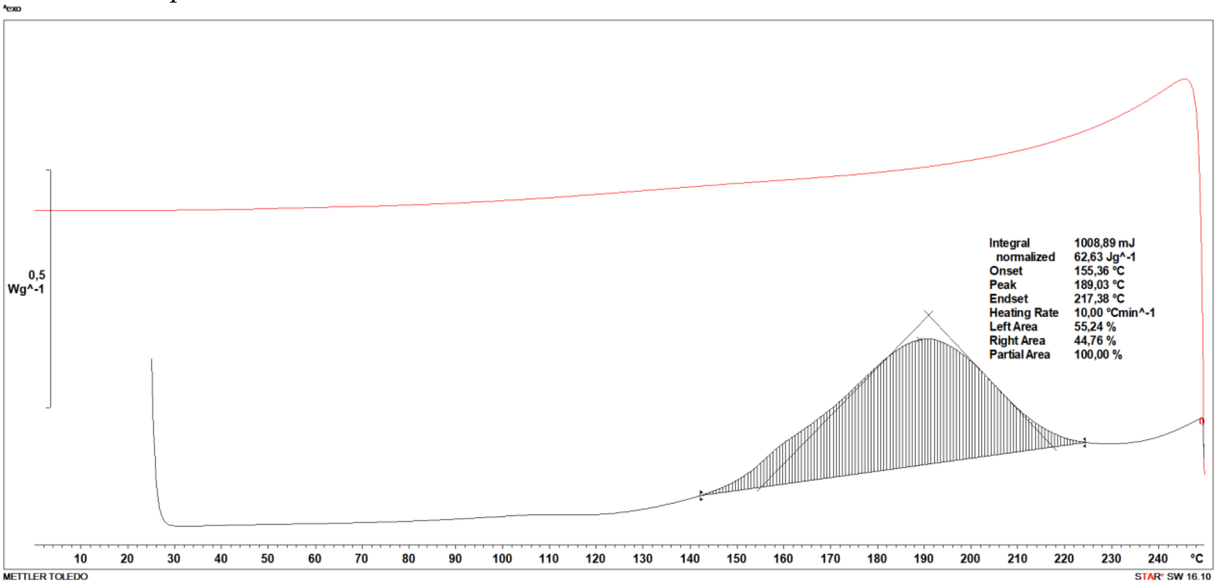

2.5. Compound **4a**

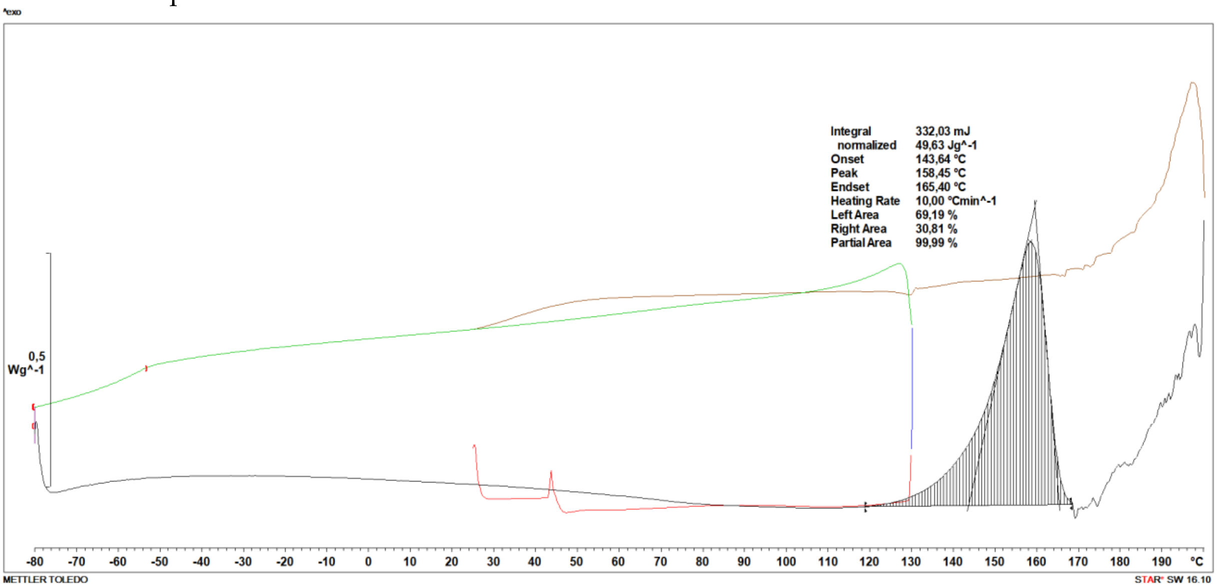

2.6. Compound **5a**

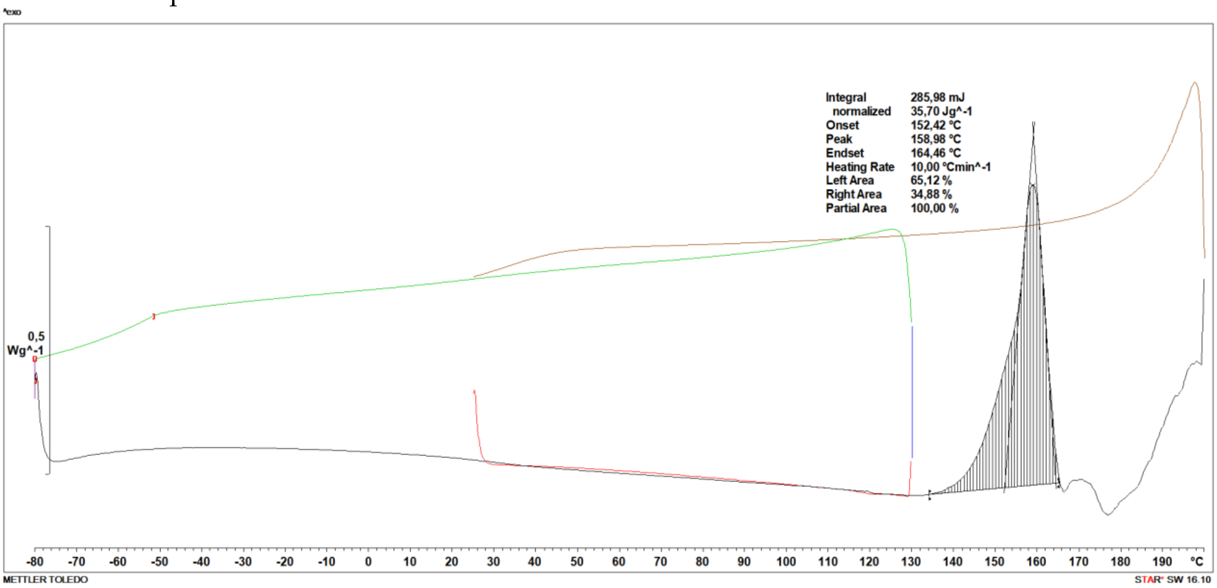

2.7. Compound **1b**

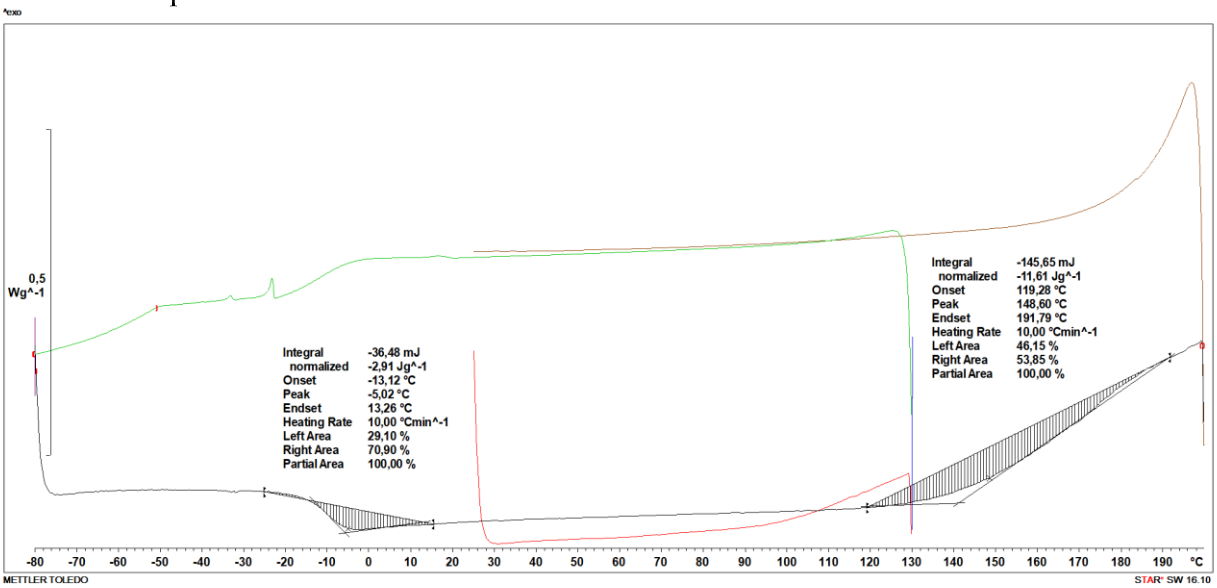

2.8. Compound **2b**

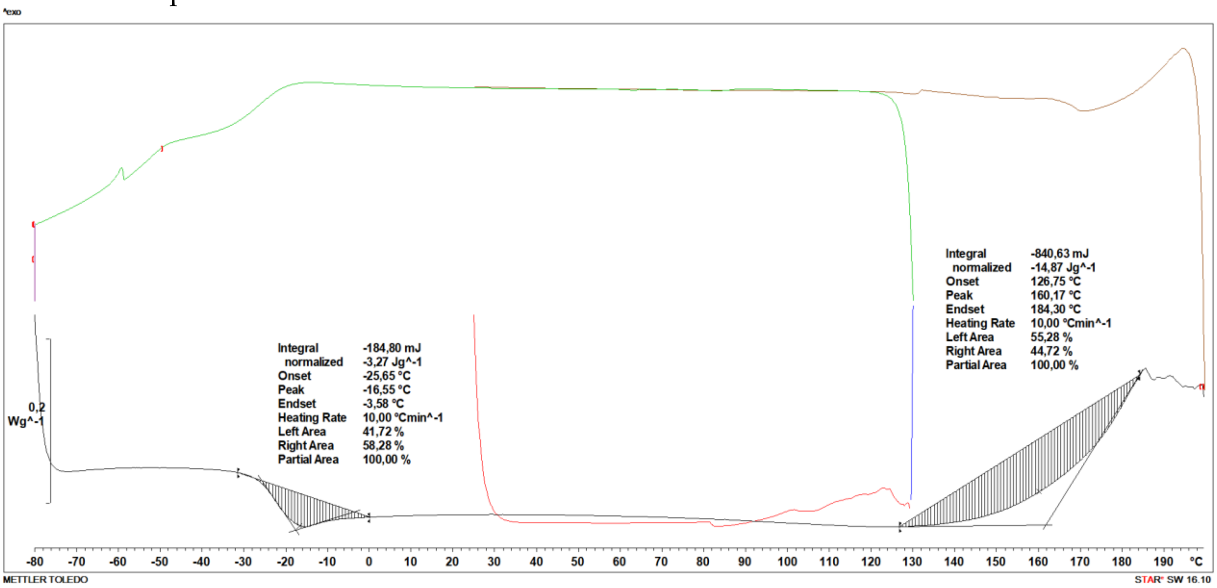

2.9. Compound **3b**

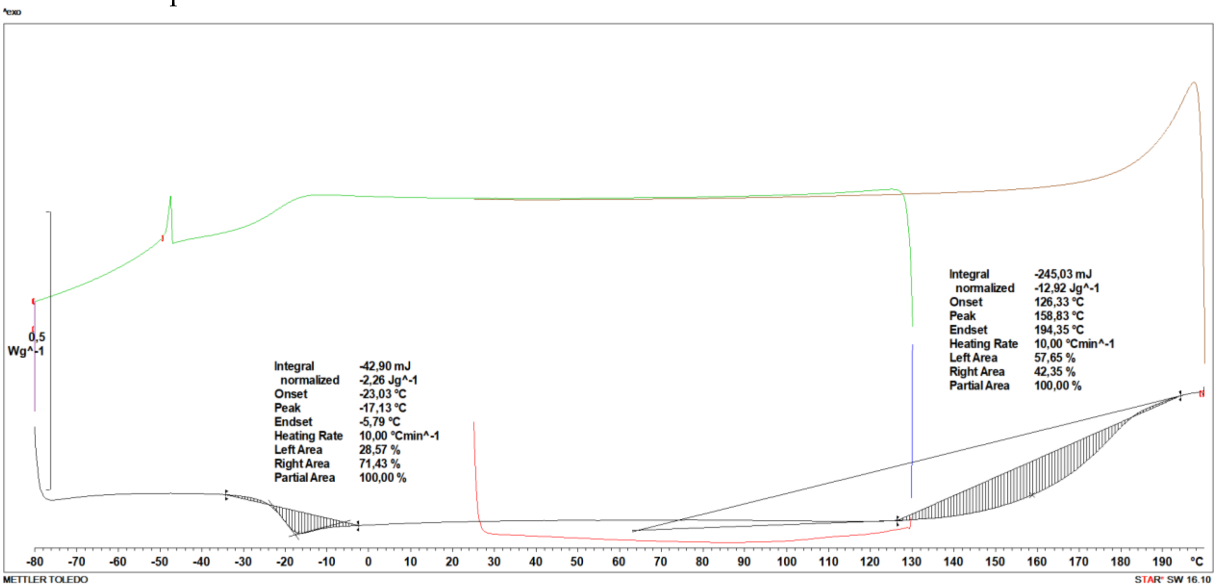

2.10. Compound **4b**

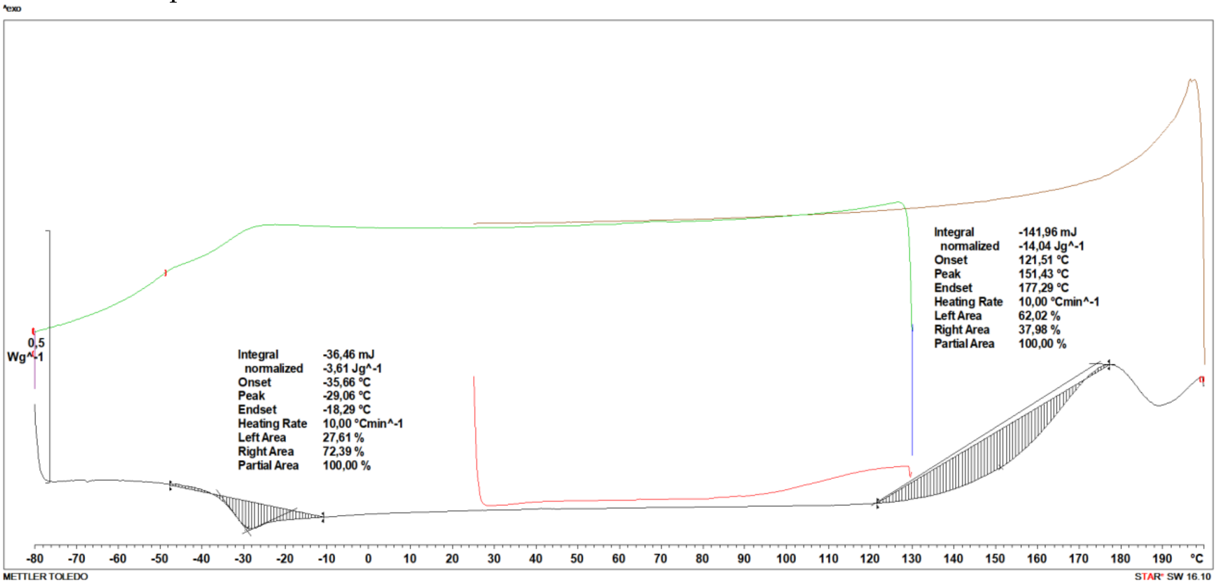

2.11. Compound **5b**

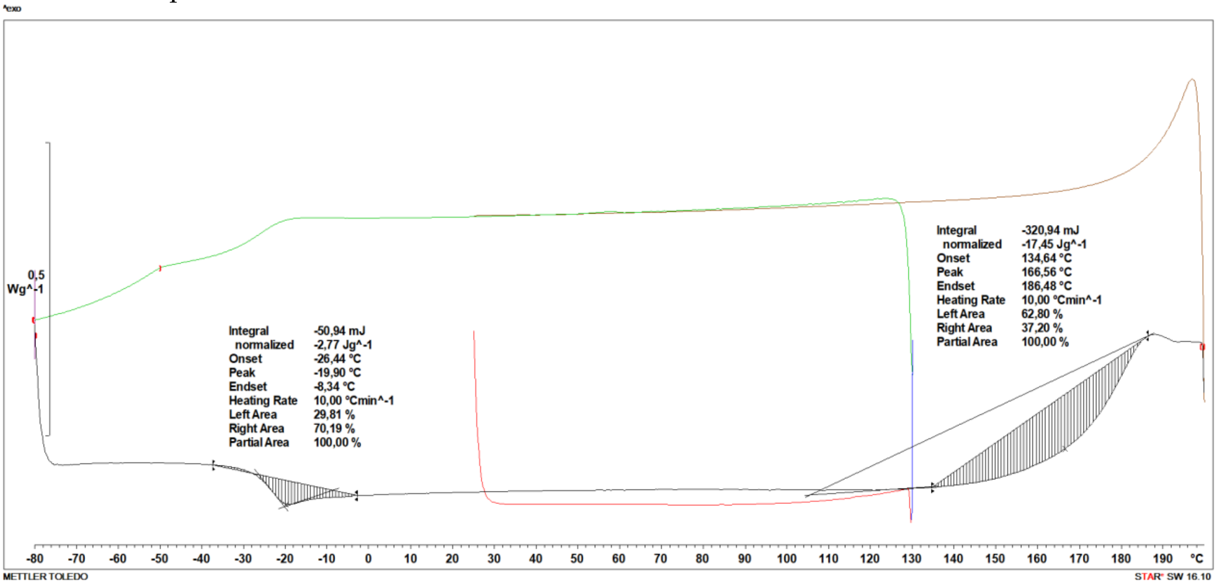

2.12. Compound **1c**

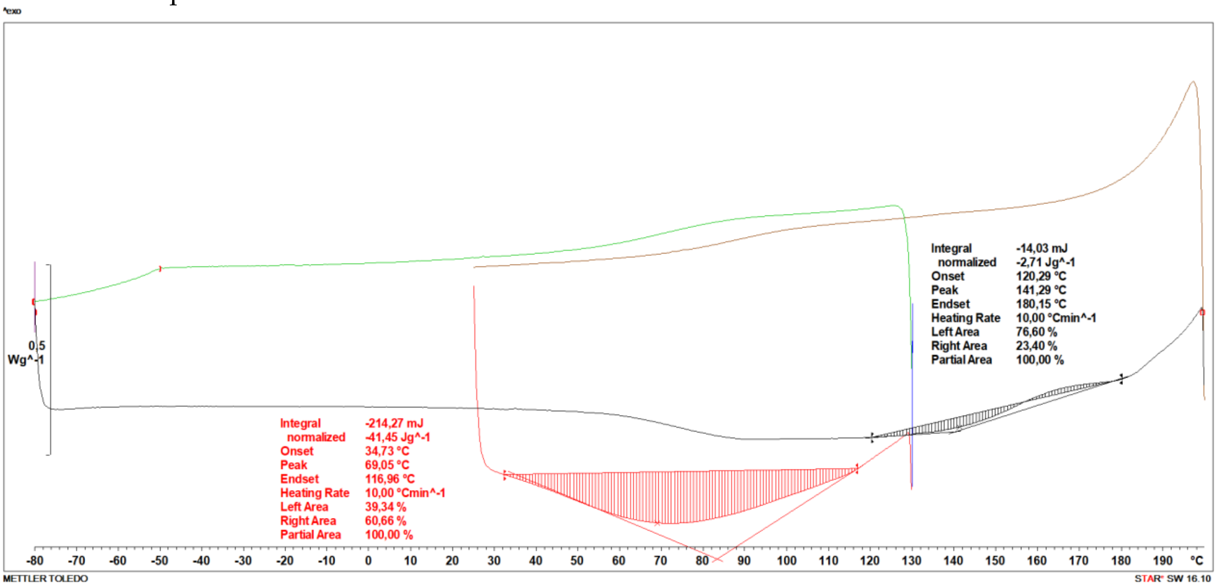

2.13. Compound **2c**

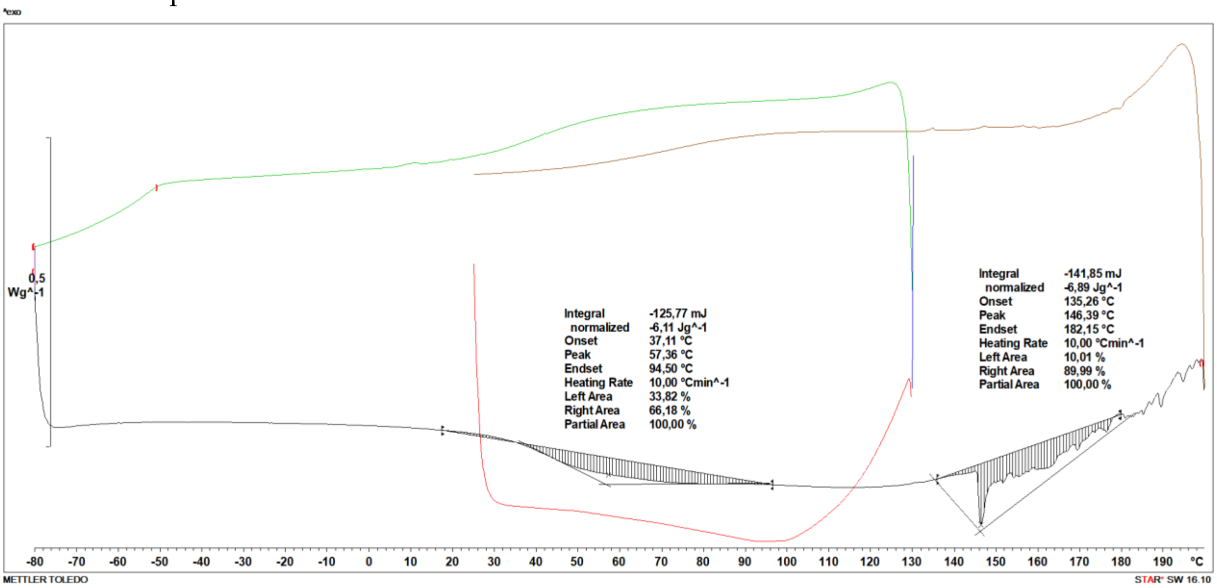

2.14. Compound **3c**

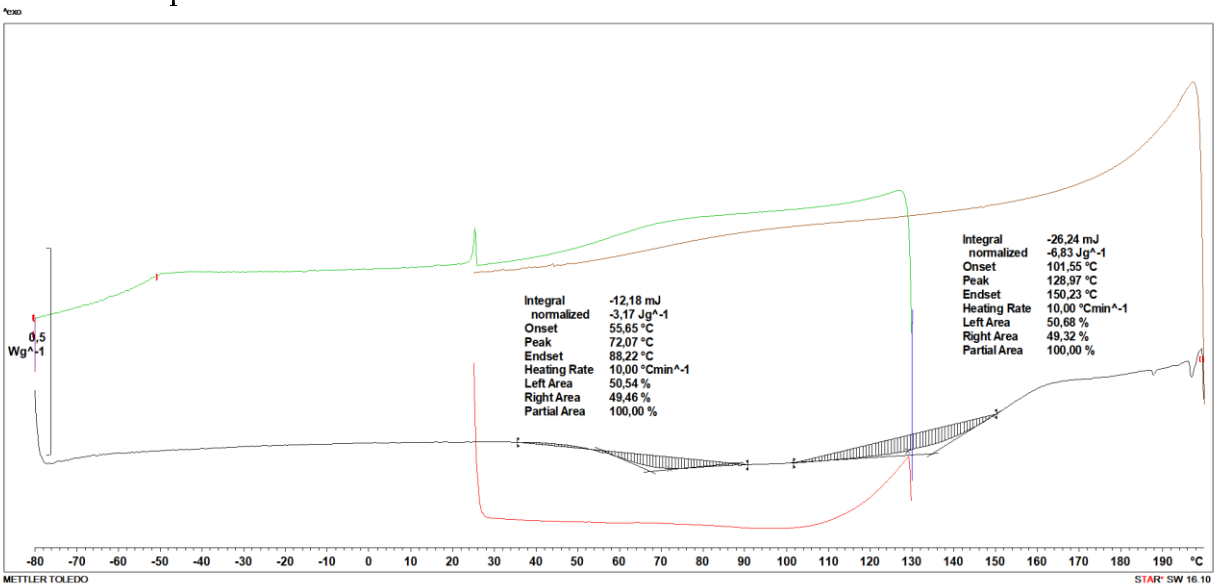

2.15. Compound **4c**

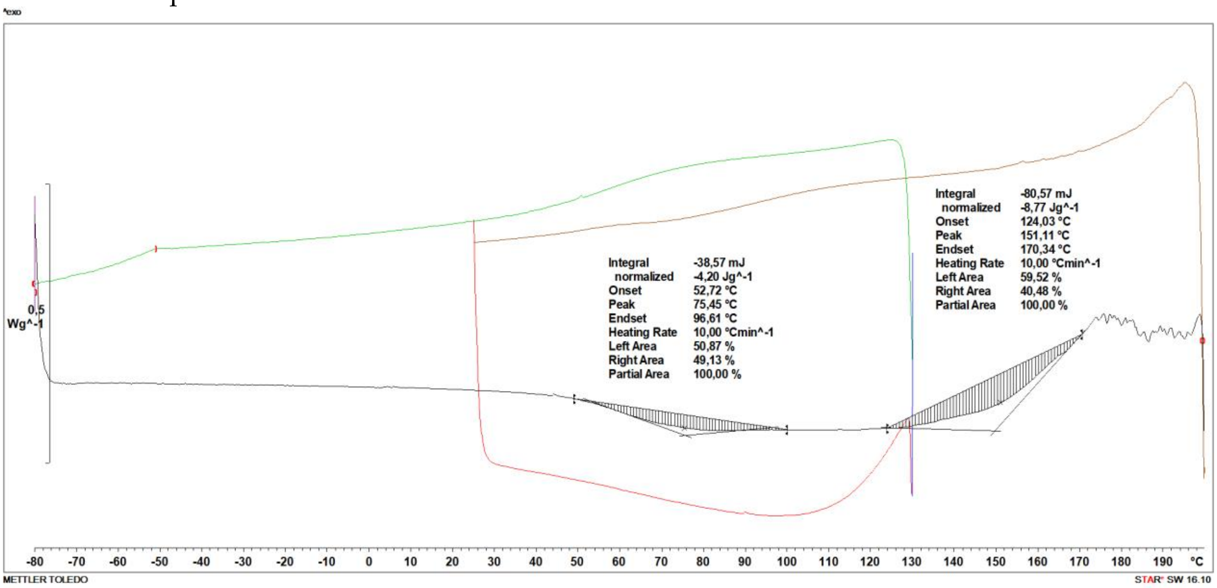

2.16. Compound **5c**

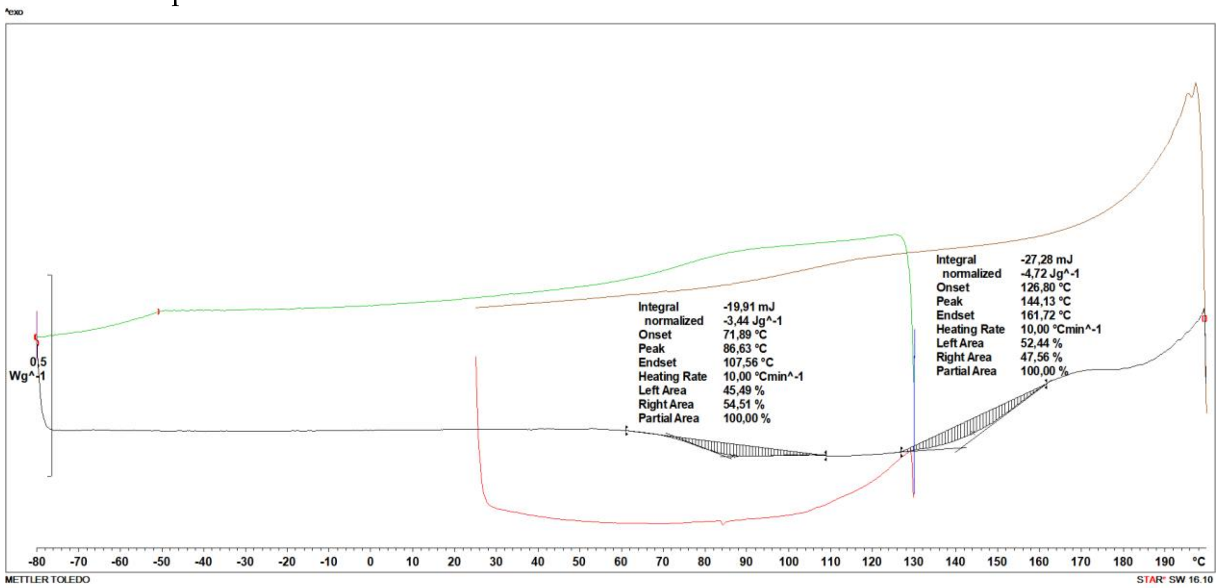

3. *DSC thermograms for curing process*

3.1. Procedure and parameters

ll of the samples were freshly prepared by mixing ionic liquids (0.2 g) with epoxy resin (5 g) in aglass vial. Each sample (5–10 mg) was analyzed using differential scanning calorimetry with a heat/cool cycle from 25 to 250 °C at 10 °C·min^{-1}. The data were collected with the same parameters as for the DSC analysis for ionic liquids [2]. For all experiments, samples in the weight range between 5 and 10 mg were used in aluminium sample pans, sealed with lids with a pin hole. An empty sample pan served as the reference.

3.2. Compound **1b**

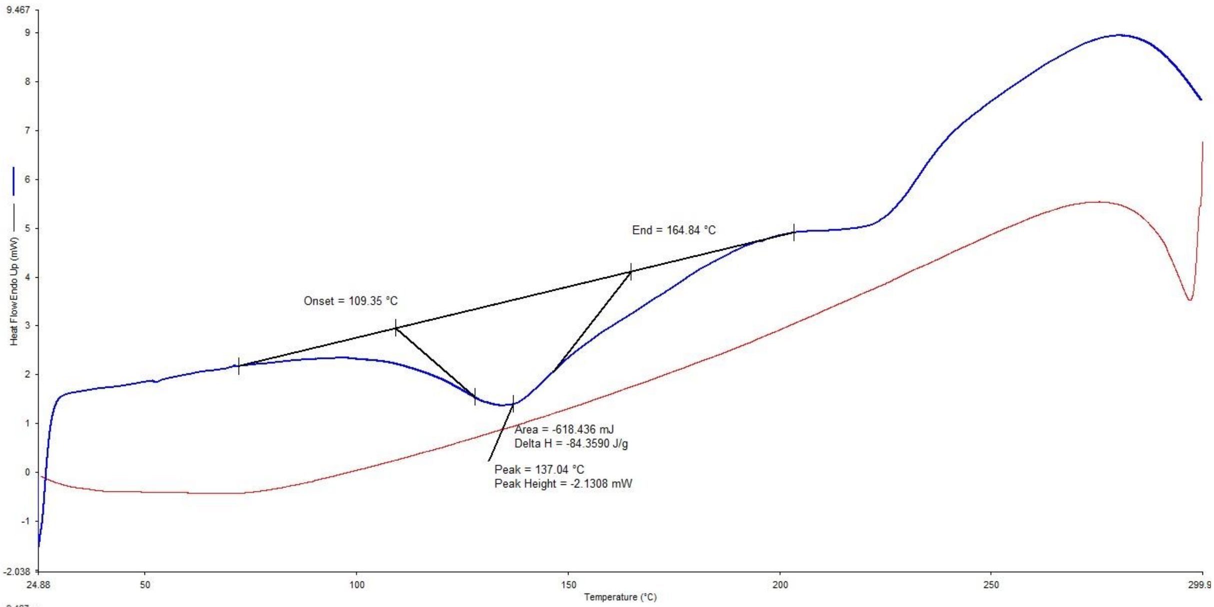

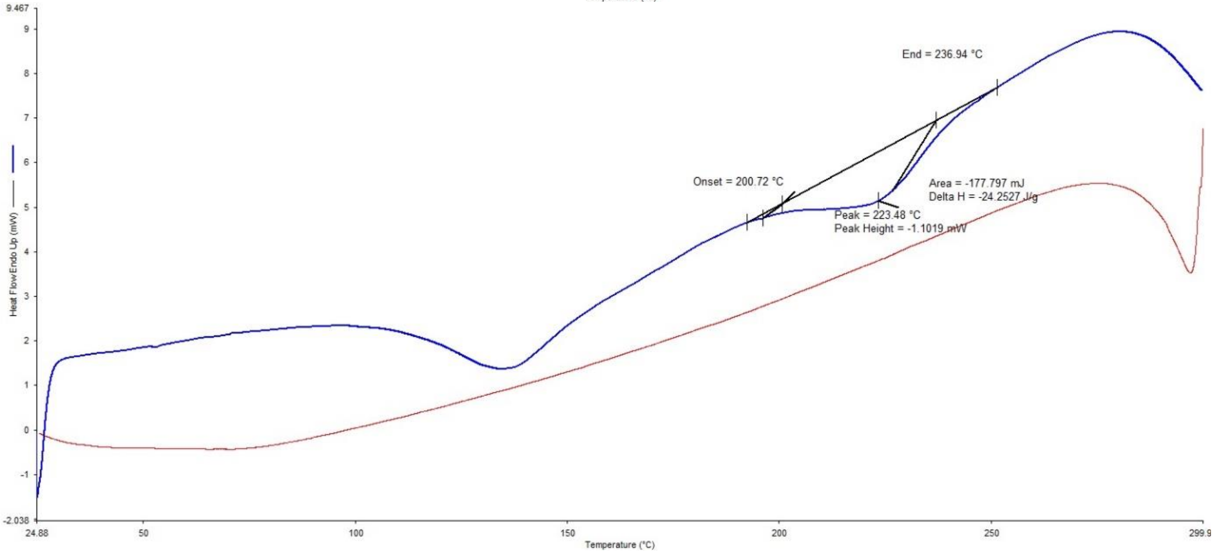

3.3. Compound **2b**

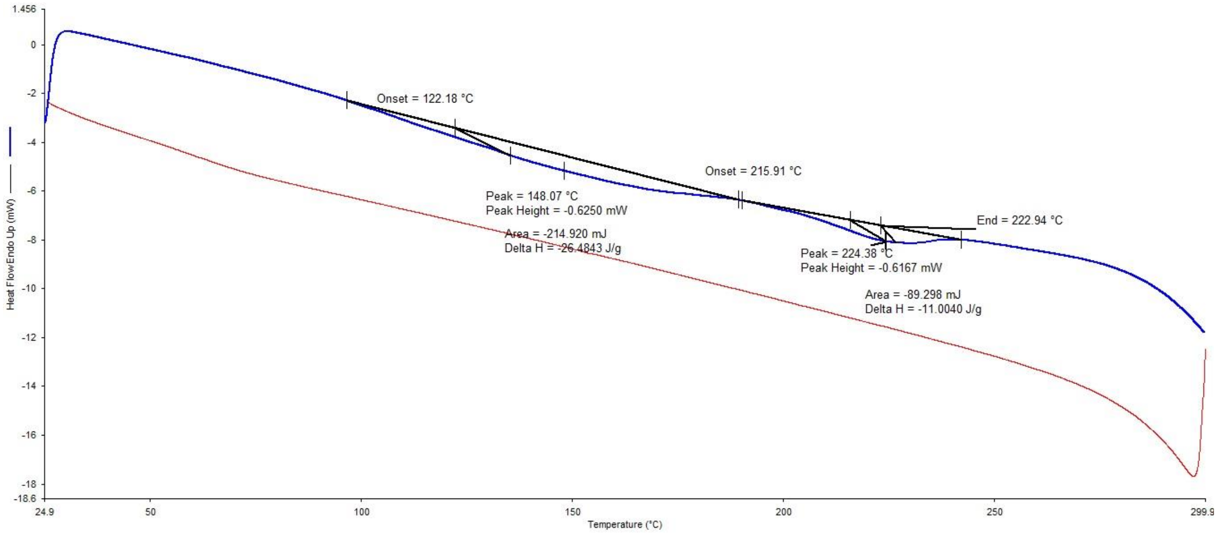

3.4. Compound **3b**

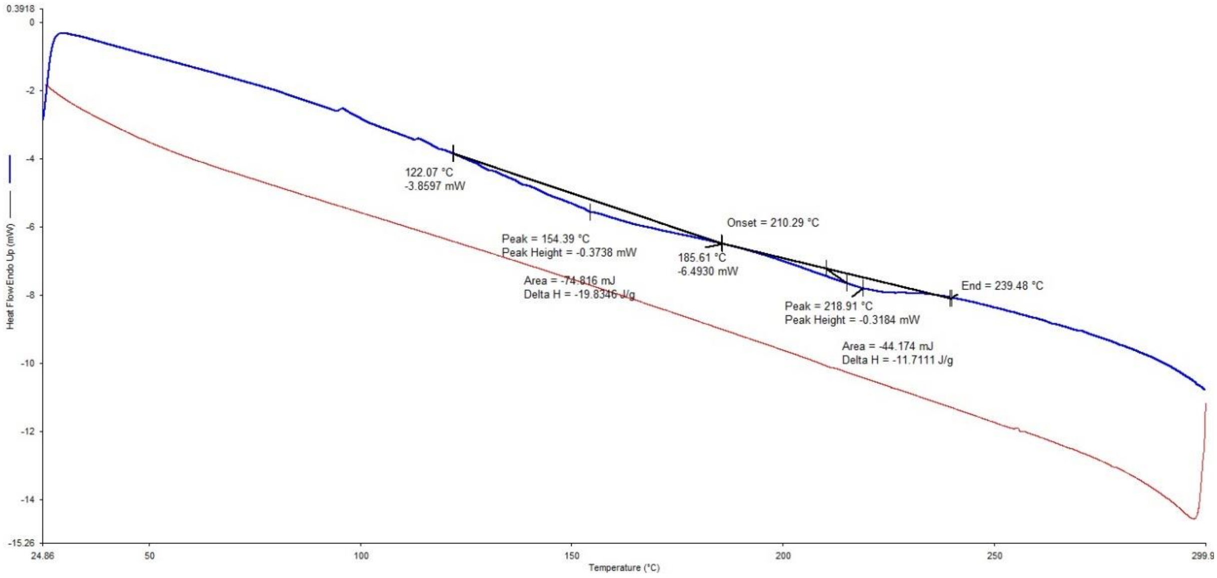

3.5. Compound **4b**

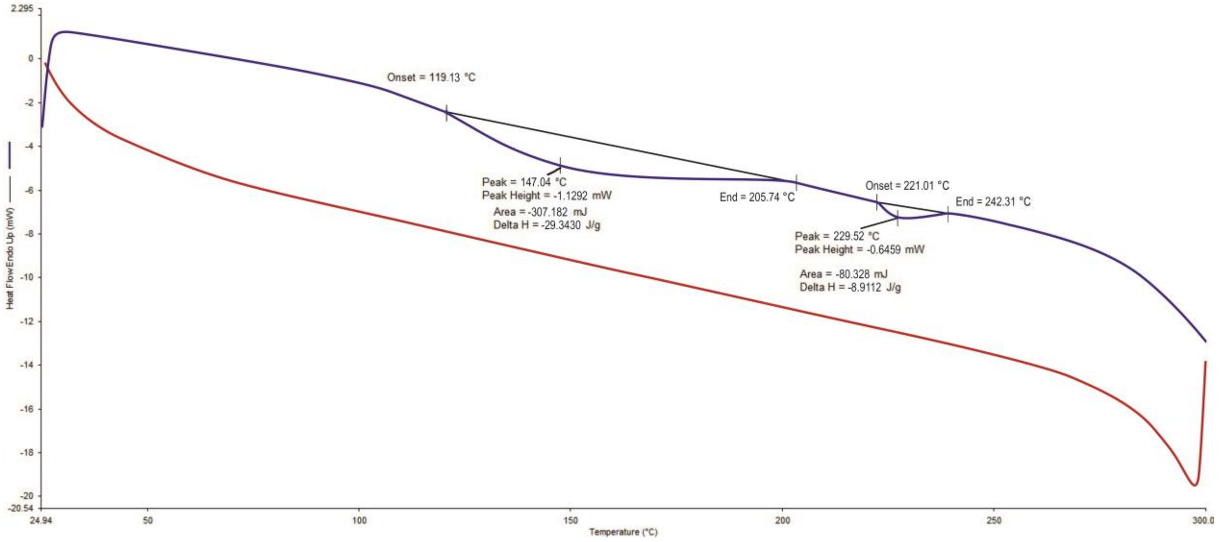

3.6. Compound **5b**

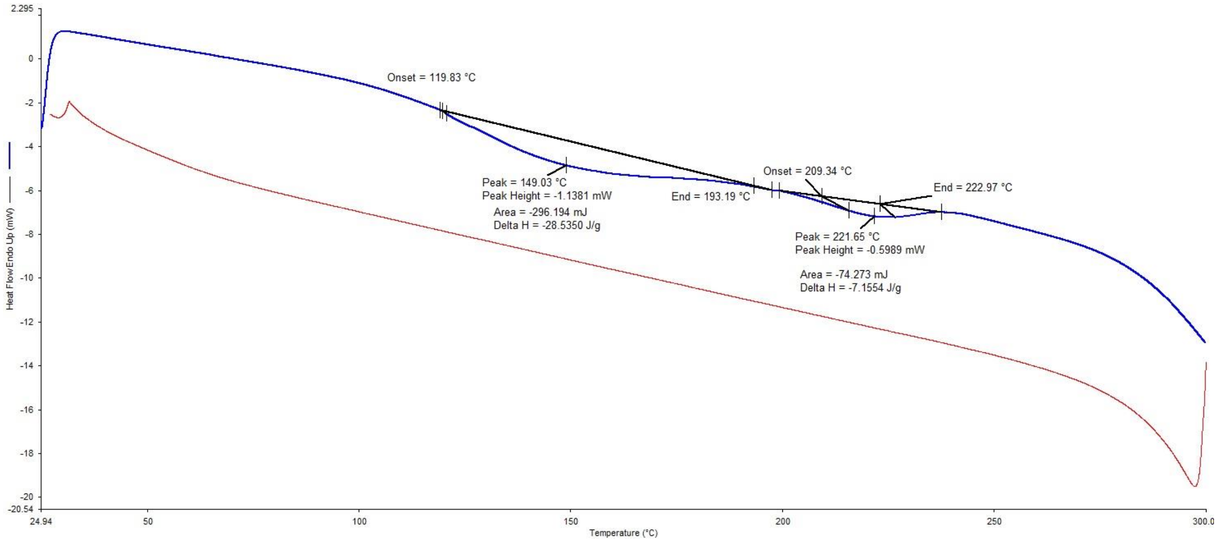

3.7. Compound **1c**

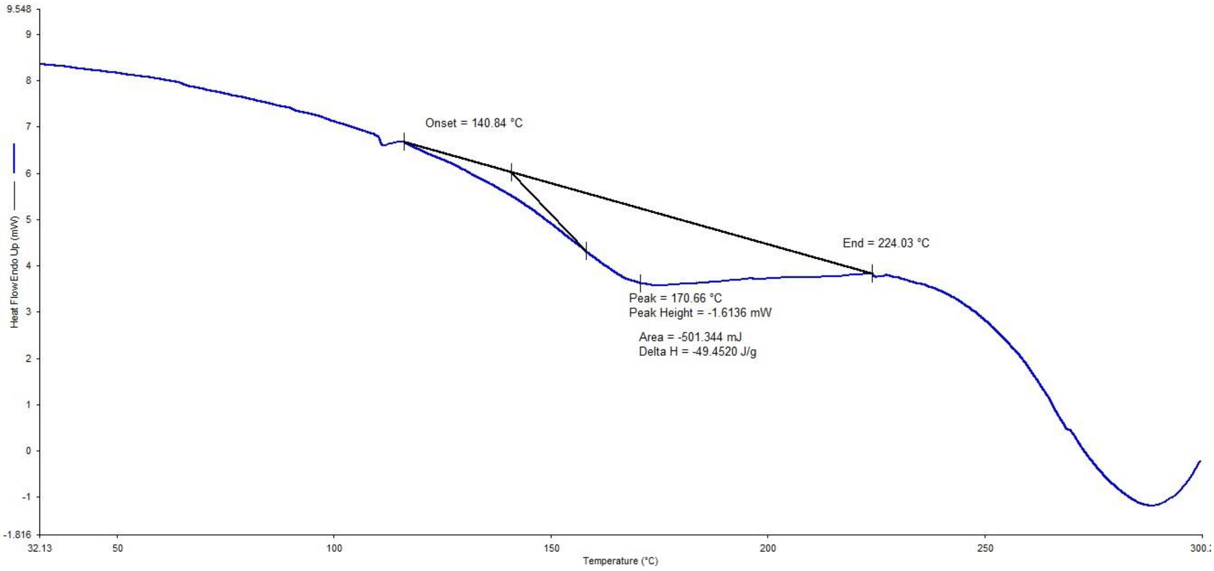

3.8. Compound **2c**

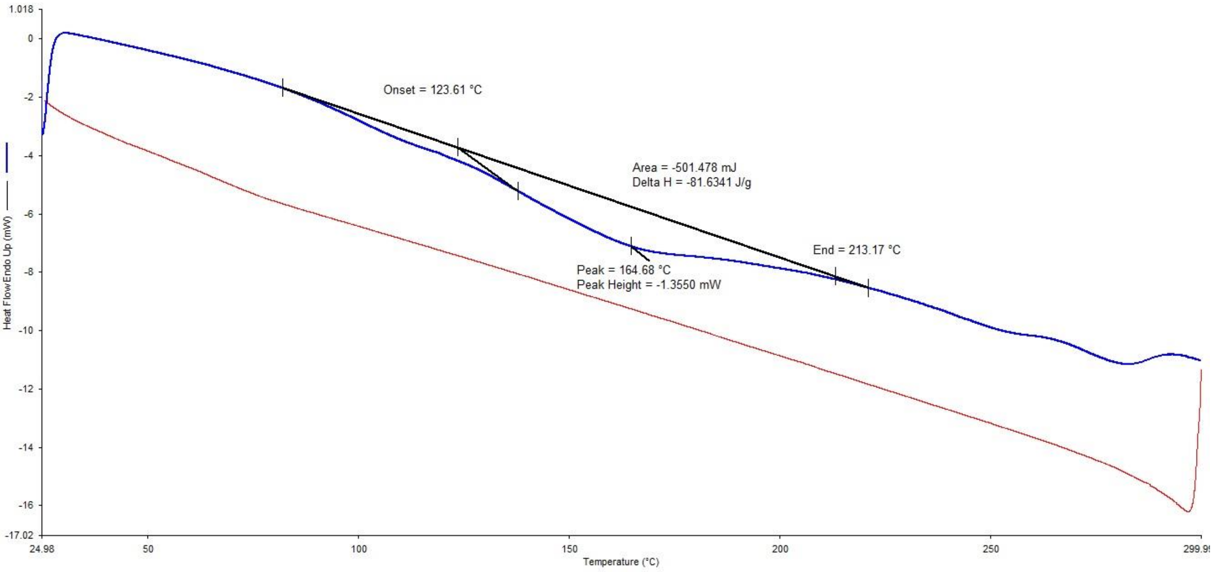

3.9. Compound **3c**

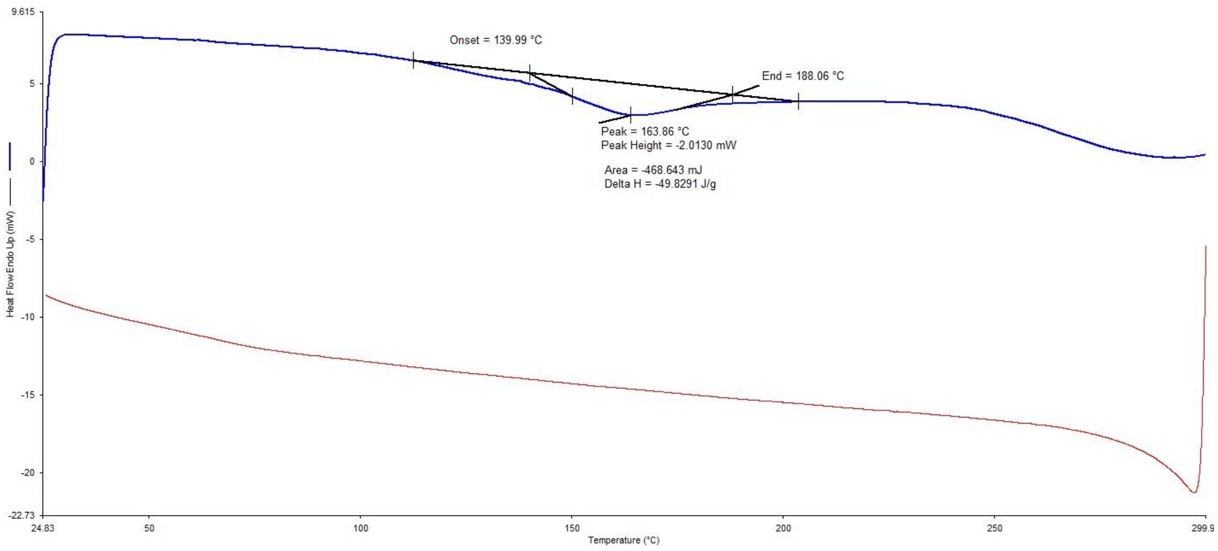

3.10. Compound **4c**

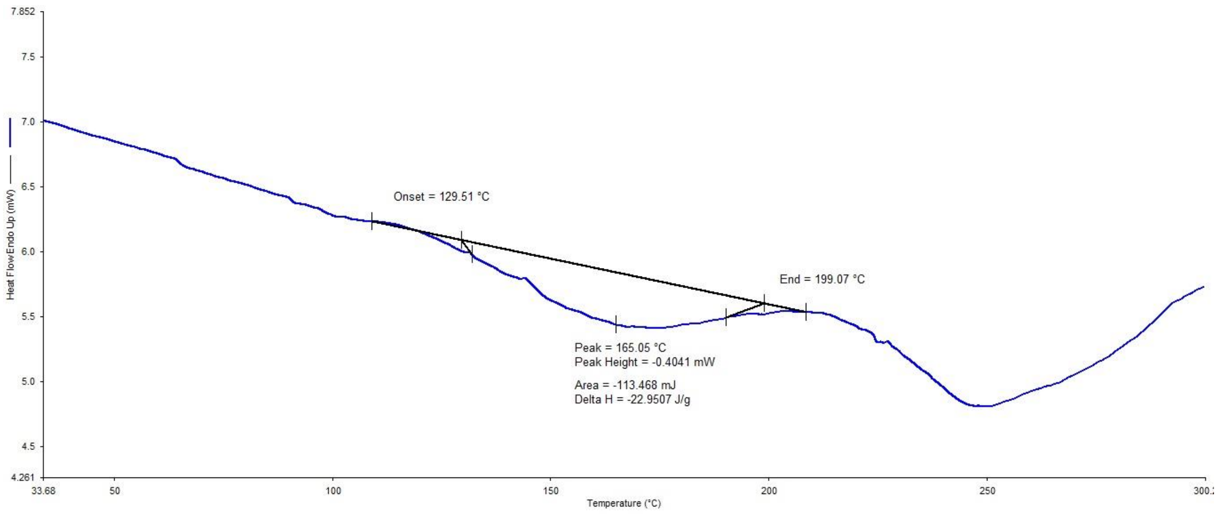

3.11. Compound **5c**

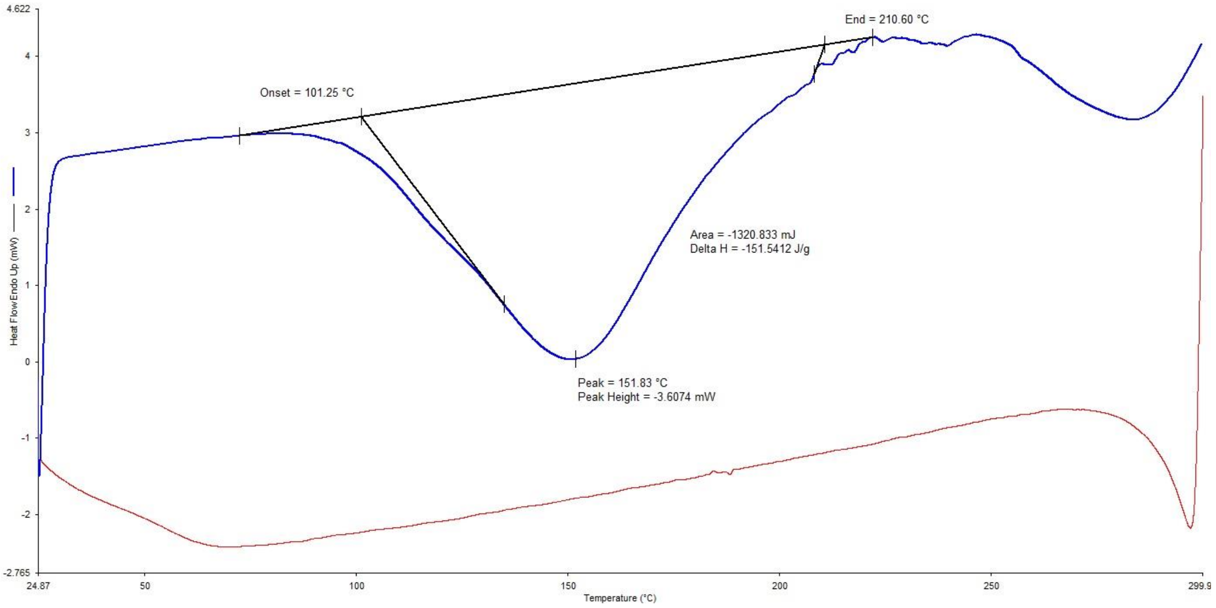

4. Microscopic pictures of cured samples

All pictures were taken with Reflecta DigiMicroscope Flex

Compound	Front	Side
**1b**	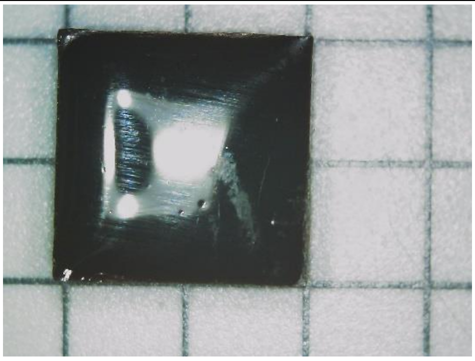	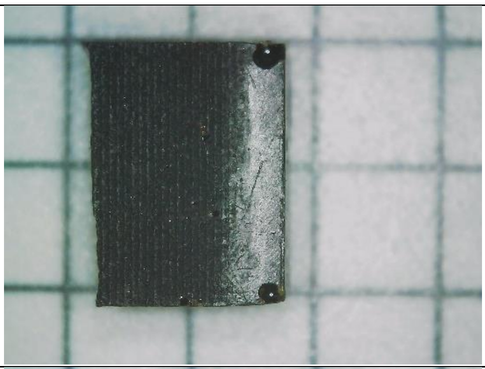
**2b**	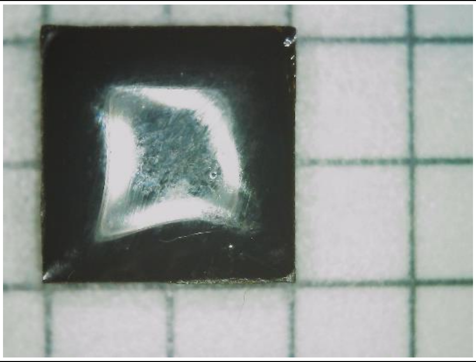	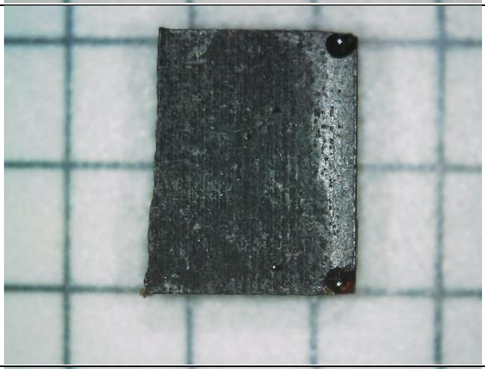
**3b**	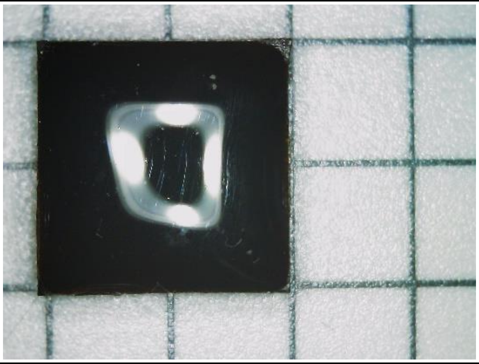	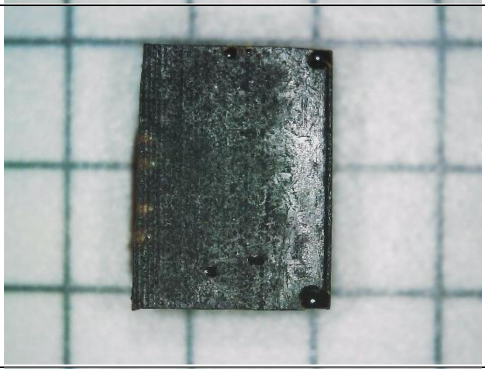
**4b**	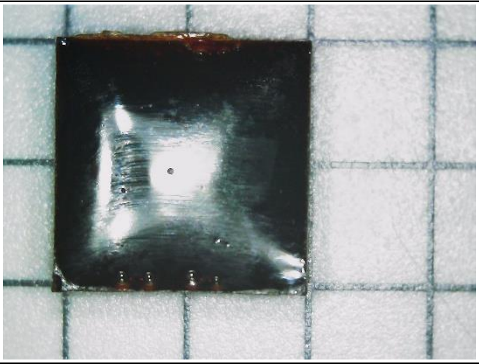	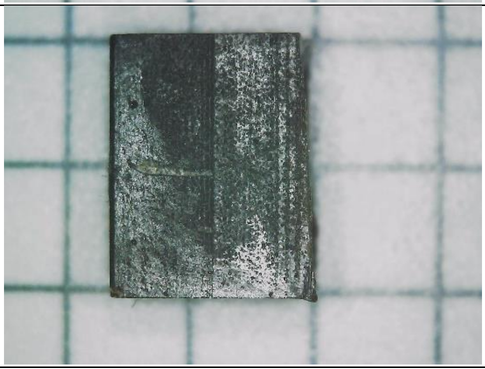

**5b**	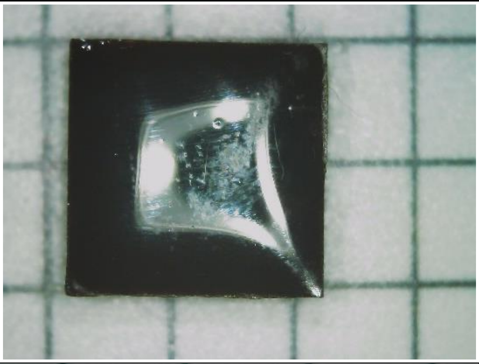	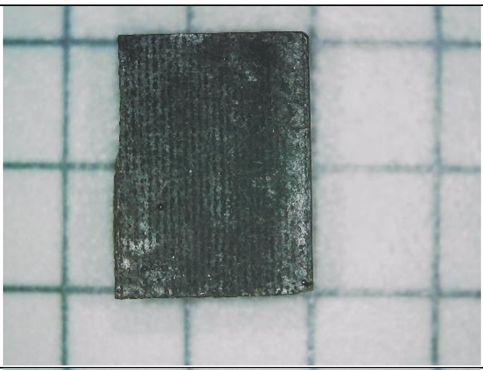
**1c**	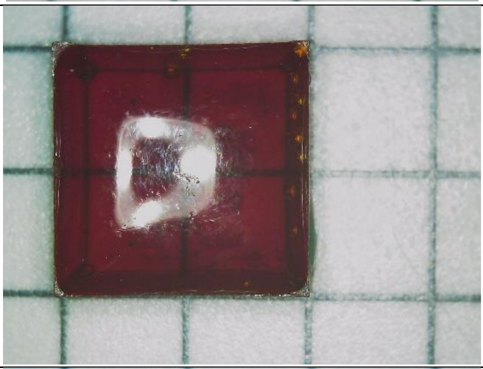	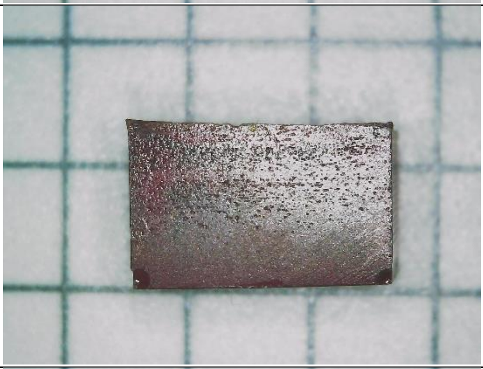
**2c**	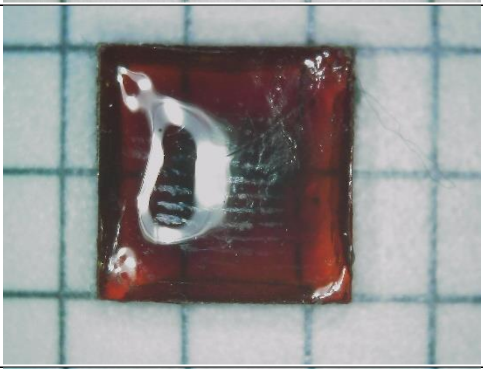	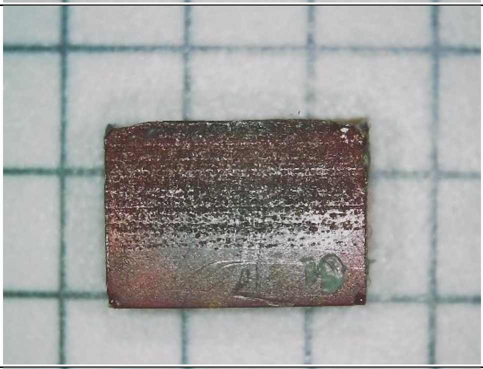
**3c**	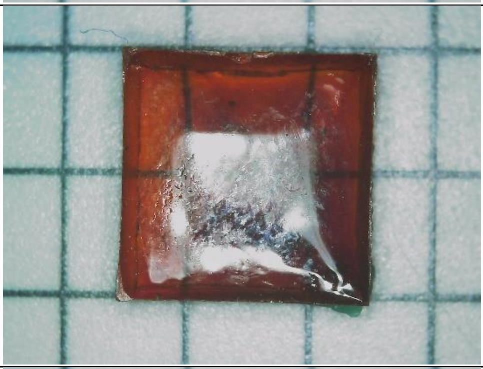	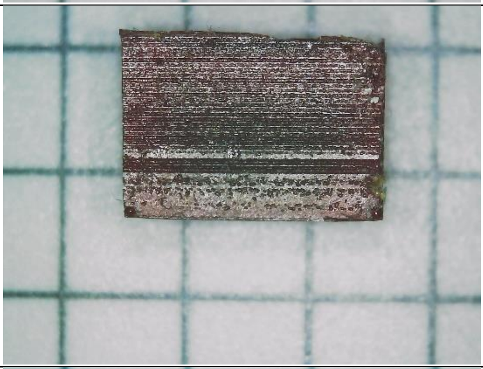
**4c**	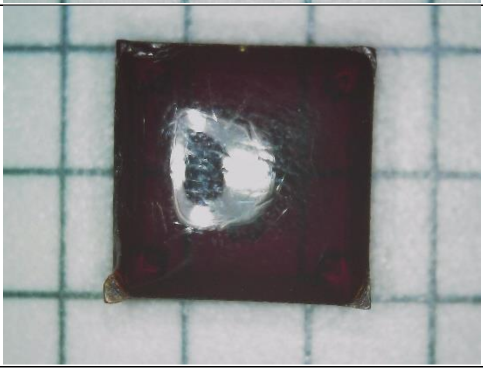	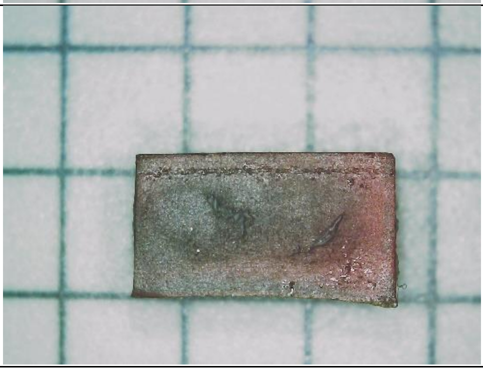

**5c**	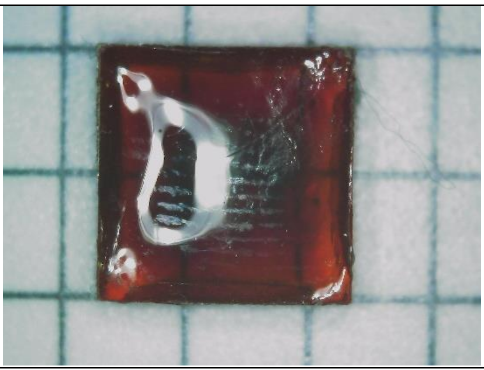	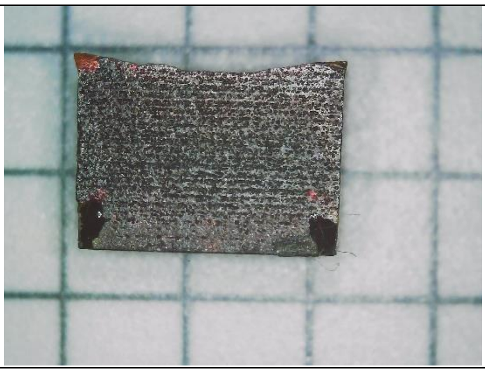

Figure S1. Microscopic pictures of cured samples.

5. References

1. Zajac, A.; Szpecht, A.; Zielinski, D.; Rola, K.; Hoppe, J.; Komorowska, K.; Smiglak, M. Synthesis and Characterization of Potentially Polymerizable Amine-Derived Ionic Liquids Bearing 4-Vinylbenzyl Group. *Journal of Molecular Liquids* **2019**, *283*, 427–439, doi:10.1016/j.molliq.2019.03.061.
2. Zielinski, D.; Szpecht, A.; Hinc, P.; Maciejewski, H.; Smiglak, M. Mono N-Alkylated DABCO-Based Ionic Liquids and Their Application as Latent Curing Agents for Epoxy Resins. *ACS Appl. Polym. Mater.* **2021**, *3*, 5481–5493, doi:10.1021/acsapm.1c00777.
